# Observation of substrate diffusion and ligand binding in enzyme crystals using high-repetition-rate mix-and-inject serial crystallography

**DOI:** 10.1107/S2052252521008125

**Published:** 2021-09-09

**Authors:** Suraj Pandey, George Calvey, Andrea M. Katz, Tek Narsingh Malla, Faisal H. M. Koua, Jose M. Martin-Garcia, Ishwor Poudyal, Jay-How Yang, Mohammad Vakili, Oleksandr Yefanov, Kara A. Zielinski, Sasa Bajt, Salah Awel, Katarina Doerner, Matthias Frank, Luca Gelisio, Rebecca Jernigan, Henry Kirkwood, Marco Kloos, Jayanath Koliyadu, Valerio Mariani, Mitchell D. Miller, Grant Mills, Garrett Nelson, Jose L. Olmos, Alireza Sadri, Tokushi Sato, Alexandra Tolstikova, Weijun Xu, Abbas Ourmazd, John C. H. Spence, Peter Schwander, Anton Barty, Henry N. Chapman, Petra Fromme, Adrian P. Mancuso, George N. Phillips, Richard Bean, Lois Pollack, Marius Schmidt

**Affiliations:** a Physics Department, University of Wisconsin-Milwaukee, 3135 North Maryland Avenue, Milwaukee, WI 53211, USA; b School of Applied and Engineering Physics, Cornell University, 254 Clark Hall, Ithaca, NY 14853, USA; c Center for Free-Electron Laser Science, Deutsches Elektronen-Synchrotron DESY, Notkestrasse 85, 22607 Hamburg, Germany; d School of Molecular Sciences and Biodesign Center for Applied Structural Discovery, Arizona State University, Tempe, AZ 85287-1604, USA; e Institute of Physical Chemistry Rocasolano, Spanish National Research Council, Calle de Serrano 119, 28006 Madrid, Spain; f European XFEL, Holzkoppel 4, 22869 Schenefeld, Germany; g Deutsches Elektronen-Synchrotron DESY, Notkestrasse 85, 22607 Hamburg, Germany; h The Hamburg Centre for Ultrafast Imaging, Luruper Chaussee 149, 22761 Hamburg, Germany; i Lawrence Livermore National Laboratory, 7000 East Avenue, Livermore, CA 94550, USA; j SLAC National Accelerator Laboratory, 2575 Sand Hill Rd, Menlo Park, California 94025, USA; k Department of BioSciences, Rice University, 6100 Main Street, Houston, TX 77005, USA; l Department of Physics, Arizona State University, Tempe, AZ 85287, USA; m Department of Bioengineering and Therapeutic Sciences, University of California San Francisco, San Francisco, CA 94158, USA; n Department of Physics, Universität Hamburg, Luruper Chaussee 149, 22761 Hamburg, Germany; o Department of Chemistry and Physics, La Trobe Institute for Molecular Science, La Trobe University, Melbourne, Victoria 3086, Australia; p Department of Chemistry, Rice University, 6100 Main Street, Houston, TX 77005, USA

**Keywords:** substrate diffusion in crystals, antibiotic resistance, β-lactamases, enzyme kinetics, irreversible inhibition, mix-and-inject serial crystallography, serial femtosecond crystallography, European X-ray Free-Electron Laser, megahertz pulse-repetition rate, protein structure determination, drug discovery, ceftriaxone, sulbactam, X-ray crystallography, enzyme mechanisms

## Abstract

Here, we illustrate what happens inside the catalytic cleft of an enzyme when substrate or ligand binds on single-millisecond timescales. The initial phase of the enzymatic cycle is observed with near-atomic resolution using the most advanced X-ray source currently available: the European XFEL (EuXFEL). The high repetition rate of the EuXFEL combined with our mix-and-inject technology enables the initial phase of ceftriaxone binding to the *Mycobacterium tuberculosis* β-lactamase to be followed using time-resolved crystallography in real time. It is shown how a diffusion coefficient in enzyme crystals can be derived directly from the X-ray data, enabling the determination of ligand and enzyme–ligand concentrations at any position in the crystal volume as a function of time. In addition, the structure of the irreversible inhibitor sulbactam bound to the enzyme at a 66 ms time delay after mixing is described. This demonstrates that the EuXFEL can be used as an important tool for biomedically relevant research.

## Abstract 2

Single-millisecond mix-and-inject experiments are presented on ligand binding to an enzyme performed at the European Free-Electron Laser with high X-ray repetition rates.

## 1. Introduction   

**Figure 1:**
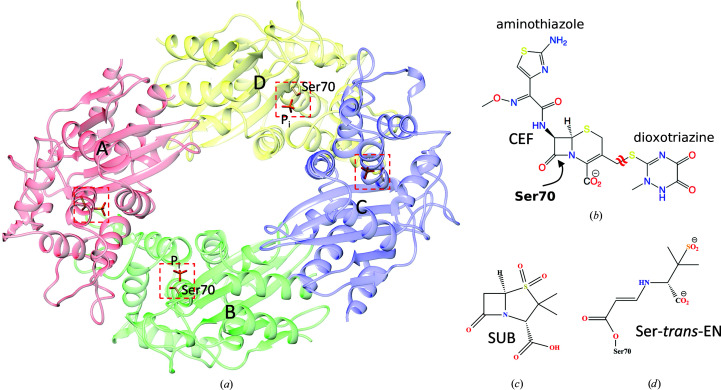
The structures of BlaC and the ligands investigated here. (*a*) Structure of BlaC in the orthorhombic crystal form. The four subunits of BlaC are shown in red (*A*), green (*B*), blue (*C*) and yellow (*D*). The red dotted box shows the position of the active site. A phosphate (P_i_) is present in all active sites. The catalytically active Ser70 is marked in subunits *B* and *D*. (*b*) The chemical structure of ceftriaxone (CEF). The leaving group (dioxotriazine; the double tilde shows the cleaved bond) and the thiazole ring are marked. (*c*) The chemical structure of sulbactam (SUB). (*d*) The covalently bound *trans*-EN. Ser70 of BlaC opens the β-lactam ring of SUB. The structure rearranges to a *trans*-enamine. This inactivates BlaC.

Combatting the rise of infectious diseases requires a collaborative and interdisciplinary approach. Structural biologists can contribute by investigating the reaction mechanisms of biomedically significant enzymes as a structural basis to develop cures for diseases. Bacterial infections with strains that are resistant to currently available antibiotics are on the rise (Cassini *et al.*, 2019 ▸). A study sponsored by the British government projected that in the near future more people will die from untreatable bacterial infections than from cancer (https://amr-review.org/). Bacterial enzymes that inactivate currently available drugs are central to antibiotic resistance (Fair & Tor, 2014 ▸), and unraveling the catalytic mechanism of these enzymes will be beneficial for the development of novel antibiotics (Imming *et al.*, 2006 ▸). β-Lactamases such as the *Mycobacterium tuberculosis* β-lactamase [BlaC; Fig. 1 ▸(*a*)] catalytically inactivate β-lactam antibiotics. β-Lactamases are responsible for the emergence of multidrug- and extensively drug-resistant bacterial strains (Smith *et al.*, 2013 ▸). Infectious diseases that could be treated with antibiotics in the past may become untreatable. This warrants the investigation of the structure and function of these enzymes.

Using time-resolved crystallography, structures of intermediates and kinetic mechanisms can be extracted simultaneously from the same set of X-ray data (Moffat, 2001 ▸; Schmidt, 2008 ▸). At free-electron lasers (XFELs) small, micrometre- and submicrometre-sized, crystals can be examined due to the immense X-ray pulse intensity (Chapman *et al.*, 2011 ▸). The microcrystals are destroyed by the pulses, and new crystals must be delivered to the X-ray interaction point in a serial fashion. This method has been termed serial femto­second crystallography (SFX; Chapman *et al.*, 2011 ▸; Boutet *et al.*, 2012 ▸). Since the XFEL pulses are of femtosecond duration, diffraction patterns are collected before the crystals suffer significant radiation damage, resulting in X-ray structures that are essentially damage-free (Lomb *et al.*, 2011 ▸; Nass, 2019 ▸; Neutze *et al.*, 2000 ▸) and are suspended in their current reaction state. Most time-resolved crystallographic experiments at XFELs are of the pump–probe type. An optical laser pulse triggers a reaction in the crystallized molecules. Structures are probed by X-ray pulses after a controlled delay (Tenboer *et al.*, 2014 ▸; Barends *et al.*, 2015 ▸; Coquelle *et al.*, 2018 ▸; Nogly *et al.*, 2018 ▸; Kern *et al.*, 2018 ▸; Pandey *et al.*, 2019 ▸; Skopintsev *et al.*, 2020 ▸; Dods *et al.*, 2021 ▸; Yun *et al.*, 2021 ▸). Due to the ultrashort nature of both X-ray and optical laser pulses, the experiments can reach subpicosecond time resolutions (Hartmann *et al.*, 2014 ▸; Barends *et al.*, 2015 ▸; Pande *et al.*, 2016 ▸; Skopintsev *et al.*, 2020 ▸). Photoactivation requires a light-sensitive cofactor, a chromophore, located in the protein to absorb the light. Light absorption must trigger a reaction that either promotes catalysis directly (Holtorf *et al.*, 1995 ▸; Li *et al.*, 2010 ▸; Sorigué *et al.*, 2017 ▸, 2021 ▸) or regulates the activity of the enzyme (Takala *et al.*, 2014 ▸; Gourinchas *et al.*, 2017 ▸; Carrillo *et al.*, 2021 ▸). Most enzymes, however, are neither activated nor regulated by light, meaning that the technique can only be directly applied in a narrow range of cases. Broader application requires great effort and chemical expertise to either engineer photoactive enzymes or to design photoactive compounds that can by soaked into, and activated in, enzyme crystals (Šrajer & Schmidt, 2017 ▸; Zaitsev-Doyle *et al.*, 2019 ▸; Mehrabi, Schulz, Dsouza *et al.*, 2019 ▸).

With the ‘mix-and-inject’ technique (Schmidt, 2013 ▸; Stagno *et al.*, 2017 ▸; Kupitz *et al.*, 2017 ▸; Olmos *et al.*, 2018 ▸; Mehrabi, Schulz, Agthe *et al.*, 2019 ▸) photoactivation is not necessary. The substrate is rapidly mixed with small enzyme crystals during sample delivery (Calvey *et al.*, 2019 ▸). Mixing occurs at a well controlled location ‘en route’ to the X-ray beam. During the time delay Δ*t*_m_ that occurs between mixing and injection, the substrate diffuses into the crystals and binds to the enzyme. The complex formed by the substrate and the enzyme then initiates the enzymatic cycle. Variation of Δ*t*_m_ allows the measurement of rate coefficients together with atomic resolution structures which can be associated with intermediate states of the protein reactions (Kupitz *et al.*, 2017 ▸; Olmos *et al.*, 2018 ▸; Mehrabi, Schulz, Agthe *et al.*, 2019 ▸). This can reveal the mechanism of enzyme action at the molecular level or the binding of a drug molecule. The combination of serial femtosecond crystallography with mixing before injection has been denoted ‘mix-and-inject serial crystallography’ (MISC; Kupitz *et al.*, 2017 ▸; Olmos *et al.*, 2018 ▸). The feasibility of MISC has previously been demonstrated with BlaC on longer millisecond timescales (Kupitz *et al.*, 2017 ▸; Olmos *et al.*, 2018 ▸). The observation of intermediate-state structures, and the maximization of the potential time resolution in both photoactivation and mix-and-inject techniques, relies on an accurately gauged start time of the reaction inside the crystals. In photoactivation experiments this requires a sufficient penetration of optical laser light into the crystal to ensure that a reaction is simultaneously triggered in a significant fraction of the molecules. In mix-and-inject experiments, the diffusion time of the substrate into the crystal may limit the ability to discriminate diffusion and kinetics, including substrate binding. To overcome this limitation, micrometre or sub­micrometre crystal sizes are required that ensure that the substrate diffuses rapidly into the crystals and the reaction is triggered swiftly and much faster than the lifetime of the reaction intermediates of interest (Schmidt, 2013 ▸).

The reaction of the cephalosporin antibiotic ceftriaxone [CEF; Fig. 1 ▸(*b*)] with BlaC is an excellent candidate for exploration with MISC. Previously, this reaction was investigated for Δ*t*_m_ of longer than 30 ms (Kupitz *et al.*, 2017 ▸; Olmos *et al.*, 2018 ▸). At 30 ms, however, the CEF binding sites in BlaC were essentially fully occupied (Olmos *et al.*, 2018 ▸), a state also reached on similar timescales for other proteins and enzymes (Stagno *et al.*, 2017 ▸; Mehrabi, Schulz, Agthe *et al.*, 2019 ▸; Ishigami *et al.*, 2019 ▸). The substrate-binding phase and the formation of the enzyme–substrate complex, however, remain elusive. Here, we aim to characterize the early phase of substrate binding with single-millisecond time delays by using the megahertz X-ray pulse-repetition rate of the European XFEL (EuXFEL; Decking *et al.*, 2020 ▸).

In addition, we aim to investigate the reaction of BlaC with an inhibitor, sulbactam [SUB; Fig. 1 ▸(*c*)], on a millisecond timescale. The biochemistry of SUB and its application in combination with β-lactam antibiotics have been described in detail elsewhere (Totir *et al.*, 2007 ▸). SUB binds to the active site of BlaC and reacts with the catalytically active serine of β-lactamases to form a covalently bound species. Most abundant is the so-called *trans*-enamine (*trans*-EN) species [Fig. 1 ▸(*d*)] that inhibits β-lactamases and helps to eliminate β-lactamase-induced antibiotic resistance. Static structures of *trans*-ENs with β-lactamases, including BlaC, have recently been characterized (Cheng *et al.*, 2020 ▸; Tassoni *et al.*, 2019 ▸), but structures of the early species that form during SUB binding remain elusive.

## 2. Methods   

### 2.1. BlaC crystals   

Platelet-shaped crystals of BlaC with approximate dimensions of 10 × 10 × 2 µm (Appendix *A* [App appa]) were produced by a stirring method on site in the XBI facility of the EuXFEL (Han *et al.*, 2021 ▸) using ammonium phosphate (AP) as described by Olmos *et al.* (2018 ▸). The crystals belonged to space group *P*2_1_ (Table 1 ▸), with four BlaC subunits in the asymmetric unit [Fig. 1 ▸(*a*)] (Olmos *et al.*, 2018 ▸). Only two subunits bind CEF in their catalytic cleft, as demonstrated previously (Olmos *et al.*, 2018 ▸). The concentration of BlaC subunits in the crystals is 15.5 m*M*, so that the concentration of active subunits is 7.8 m*M*. When this concentration is matched by substrate, the substrate concentration is called ‘stoichiometric’ in the following description.

### 2.2. Data collection at the EuXFEL   

**Figure 2:**
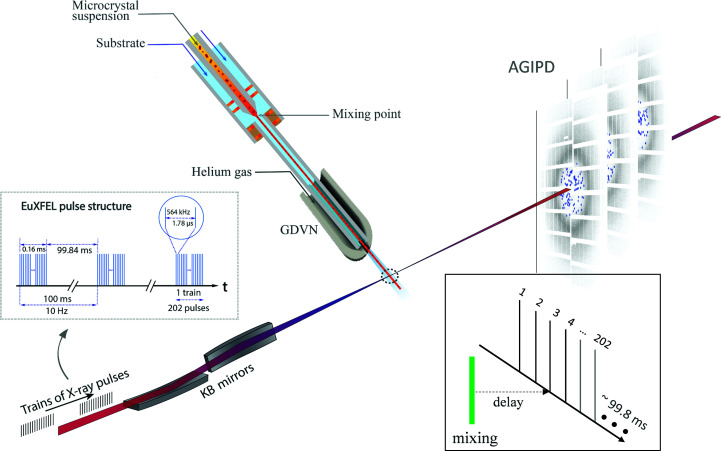
Experimental setup at the European XFEL. BlaC microcrystals are mixed with substrate and injected into the X-ray interaction region (dotted circle) after a delay determined by the distance between the mixing region and the X-rays, the capillary width and the flow rate. Diffusion of substrate into the crystals occurs during this time. The mixture is probed by trains of X-ray pulses. The trains repeat ten times per second. Pulses within the trains repeat at 564 kHz, hence the pulses are spaced by 1.78 µs. 202 pulses were in each train for this experiment. The AGIPD collects the diffraction patterns and reads them out for further analysis. Inset: data collection. With a selected injector geometry and flow rate, the delay is fixed by the distance of the mixing region from the X-ray interaction region. All pulses in all trains (here pulse 3) probe the same time delay. The EuXFEL pulse structure is most efficiently used.

The platelets were mixed with ceftriaxone [CEF; Fig. 1 ▸(*b*); molecular mass 554.6 g mol^−1^; 200 m*M* in 0.8 *M* AP] or sulbactam (SUB) inhibitor [Fig. 1 ▸(*c*), molecular mass 223.2 g mol^−1^, 100 m*M* in 0.8 *M* AP] using optimized mixing injectors (Calvey *et al.*, 2019 ▸) which were adapted to operate at the SPB/SFX instrument (Mancuso, 2019 ▸) of the EuXFEL. Flow rates and mixer geometries are shown in Table 2 ▸. The mixture was intercepted after a delay Δ*t*_m_ by X-ray pulses from the EuXFEL. The EuXFEL delivers X-ray pulses in pulse trains that repeat ten times per second (Fig. 2 ▸). Each train contained 202 X-ray pulses with approximately 40 fs full-width at half-maximum (FWHM) pulse duration and about 1.5 mJ pulse energy. The pulse-repetition rate within a pulse train was 564 kHz, a reduction from the possible 4.5 MHz to avoid pristine, upstream jet volumes being affected by previous X-ray pulses (Yefanov *et al.*, 2019 ▸; Pandey *et al.*, 2020 ▸; Grünbein *et al.*, 2021 ▸). Given a flow rate of about 80 µl min^−1^ (Table 2 ▸) and an assumed jet diameter of 8 µm, the jet advances 26.5 m in a second. With a 564 kHz pulse rate the jet is intercepted every 47 µm, which is much larger than the gap (∼20 µm; Wiedorn, Oberthür *et al.*, 2018 ▸) in the jet produced by the intense X-ray pulse. The X-ray beam size at the jet position was ∼3 µm. The design of the mixers allowed us to recapture the record (Mehrabi, Schulz, Agthe *et al.*, 2019 ▸) for the shortest MISC time point, while maintaining the high jet speed necessary for the 564 kHz measurements (Wiedorn, Oberthür *et al.*, 2018 ▸). Reference data were obtained by mixing with water.

Diffraction patterns (DPs) were collected using the Adaptive Gain Integrating Pixel Detector (AGIPD; Allahgholi *et al.*, 2019 ▸) operating at a 565 kHz frame rate. The experiment was monitored using *OnDA* (Mariani *et al.*, 2016 ▸), which is designed to estimate hit rates and spatial resolution in real time. DPs with Bragg reflections were selected by *Cheetah* (Barty *et al.*, 2014 ▸) and indexed, integrated, scaled and merged by *CrystFEL* (White *et al.*, 2016 ▸) in a manner consistent with previous work (Pandey *et al.*, 2020 ▸). In brief, diffraction images with Bragg reflections were found by *Cheetah* (peakfinder8, minSNR=8, minADC=200, minPix=1, minPeaks=25) using the calibration process described by Wiedorn, Awel *et al.* (2018 ▸). Careful masking of shadowed and unreliable regions of the detector was performed on a run-by-run basis (Appendix *B* [App appb]). Independent masks were used for peak finding to avoid false hits, for example due to ice formation. Indexing was performed with *CrystFEL* (version 0.9.0) using the indexing package *XGANDALF* (Gevorkov *et al.*, 2019 ▸) with the following parameters: peaks=peakfinder8, Min-SNR=5, Min-pixel-count=1, Threshold=400. The detector geometry was refined using *geoptimiser* (Yefanov *et al.*, 2015 ▸). Merging and scaling of the Bragg peak intensities were performed using the *partialator* program from *CrystFEL*. To avoid the integration of noise for weakly scattering patterns, reflections were included up to 1.0 nm^−1^ above a conservative resolution estimate for each crystal (--push-res=1.0). Hit rates and indexing rates were stable in the order of 1.0% and 70%, respectively, irrespective of the pulse index in the train (Appendix *A* [App appa]). The lower hit rate is a consequence of diluting the crystalline slurry with the ligand/substrate. It has been shown that the X-ray pulse position in the train has no effect on the structure (Yefanov *et al.*, 2019 ▸). Structure-factor amplitudes were generated from the measured intensities using programs from the *CCP*4 software suite (Winn *et al.*, 2011 ▸). Data-collection statistics are shown in Table 1 ▸.

As a control, and to investigate the result of the complete reaction of BlaC with SUB in the platelet crystal form, macroscopic crystals were grown in sitting drops (10 µl BlaC at 45 mg ml^−1^ mixed in a 1:1 ratio with 2.1 *M* AP pH 4.1). Crystals grew within three days. The crystals were soaked for 3 h in a cryobuffer consisting of 2 *M* AP, 20% glycerol and 100 m*M* SUB. The crystals were cooled in liquid nitrogen. Data were collected on beamline ID-19 of the Structural Biology Center, Advanced Photon Source, Argonne National Laboratory. Data were processed to 2.7 Å using *HKL*-3000 (Minor *et al.*, 2006 ▸). Details will be presented elsewhere.

### 2.3. Difference-map calculation and structure determination   

The structures of BlaC and the BlaC–CEF complexes were determined as described previously (Kupitz *et al.*, 2017 ▸; Olmos *et al.*, 2018 ▸). Since the unit-cell constants change substantially after mixing (Table 1 ▸), isomorphous difference maps cannot be calculated and OMIT difference maps (DED_omit_) were used. An initial BlaC model, PDB (Berman *et al.*, 2002 ▸) entry 6b5x (Olmos *et al.*, 2018 ▸), was refined against the structure-factor amplitudes |*F*_o_(*t*)|_CEF_ collected at a particular Δ*t*_m_. The content of the active sites (water and phosphate) was removed during the refinement. From the refined model amplitudes, |*F*_c_| were determined. From the amplitudes (and the phases obtained from the refined model), weighted *m*|*F*_o_(*t*)|_CEF_ − *D*|*F*_c_| OMIT maps (DED_omit_) were calculated. Polder difference maps (Liebschner *et al.*, 2017 ▸; DED_Polder_) were calculated to display weak difference electron-density features. The CEF was modeled in the polder maps. To refine the structure and to determine the fractional concentration of both P_i_ and CEF, grouped occupancy refinement was performed using *Phenix* (Liebschner *et al.*, 2019 ▸). CEF was flagged together with P_i_ as a pair of molecules occupying the same space. The positions and *B* factors of all atoms as well as the occupancies of P_i_ and CEF were refined simultaneously. As a check, the sum of the occupancies of the flagged molecules should not deviate too much from unity.

Initial structures of the BlaC–SUB complexes were determined by inspecting both isomorphous and OMIT difference maps. The OMIT map was calculated in a similar way as described above except that amplitudes |*F*_o_(*t*)|_SUB_ were used. A weighted DED_iso_ map was calculated from difference amplitudes *w*[|*F*_o_(*t*)|_SUB_ − |*F*_o_|_WAT_], where the reference amplitudes |*F*_o_|_WAT_ were obtained by mixing with water. The weighting factor was calculated as described previously for photoactive yellow protein (Ren *et al.*, 2001 ▸; Pandey *et al.*, 2020 ▸) and for the needle crystal form of BlaC (Olmos *et al.*, 2018 ▸). The *trans*-EN and SUB molecules were inserted into the OMIT map. The positions and orientations were cross-examined to agree with the DED_iso_ map. The complexes were refined using *REFMAC* (Murshudov *et al.*, 2011 ▸) against the |*F*_o_(*t*)|_SUB_ amplitudes. The refinement statistics for the BlaC–CEF and BlaC–SUB complexes are shown in Table 3 ▸.

### 2.4. Binding kinetics of CEF   

Refined occupancies are fitted by functions that account for saturation of CEF and decline of P_i_,

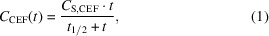

and

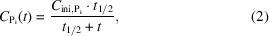

respectively. 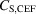 and 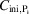 are the occupancy of CEF after a long time (at saturation) and the initial occupancy of P_i_, respectively. The constant *t*_1/2_ is either the time taken to reach half saturation of CEF or denotes the time when P_i_ has declined to half its initial concentration. The initial occupancy of P_i_ was set as 1.0, but the final occupancy of CEF was not constrained to 1.0 to account for a more realistic scenario.

### 2.5. The diffusion coefficient of CEF in the BlaC platelets   

The occupancy of CEF bound noncovalently to the active center of BlaC was calculated with typical sized 10 × 10 × 2 µm (platelet-like) crystals (Appendix *A* [App appa]) by varying the diffusion coefficient *D* for the CEF in crystals until agreement with the experiment was achieved. The crystal was divided into 20 voxels along each direction and 20 time intervals were used. For each time interval, the concentration of the CEF substrate (*C*_CEF_) in each of the 8000 voxels in the crystal was determined using the known solution to Fick’s second law for a rectangular volume, represented by the first 20 modes (Schmidt, 2013 ▸, 2020 ▸; Carslaw & Jaeger, 1959 ▸),

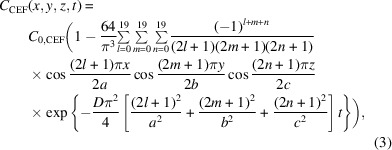

where *l*, *m*, *n* are integer numbers that define the modes. *x*, *y* and *z* are coordinates within the crystal that extend from −*a* to +*a*, −*b* to +*b* and −*c* to *c*, where *a*, *b*, *c* are the half edge lengths of the platelet-shaped BlaC crystals. *D* is the diffusion coefficient, *t* is the time after mixing and *C*_0,CEF_ is the outside CEF concentration, which was set to 150 m*M*. This analytical approach to diffusion is strictly speaking only valid in the absence of substrate binding. However, here the substrate concentration (∼150 m*M*) is much higher than the concentration of the subunits that bind the CEF (7.8 m*M*). The concentration of substrate in the crystals increases rapidly to values much higher than the stoichiometric concentration. At saturation, the ES concentration is only 5.2% of that of the substrate. In addition, the speed (rate) of substrate binding is low until sufficient substrate is present. Accordingly, substrate binding is a small perturbation of the free CEF concentration on all but the very shortest timescales.

**Figure 3:**
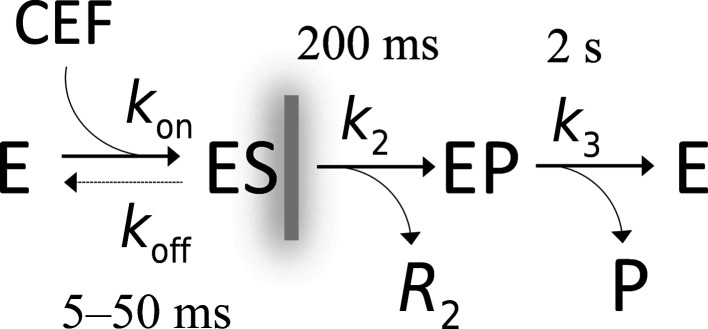
Simplified enzymatic cycle of, and the timescales associated with, the reaction of BlaC with CEF. CEF is delivered to the crystals by diffusion. It noncovalently binds to the free BlaC enzyme (E) to form the enzyme–substrate complex (ES). The acyl intermediate EP, which is covalently bound to Ser70, is formed within ∼200 ms. The leaving group *R*_2_ is cleaved off the CEF. The modified CEF (EP) is hydrolyzed and released as product (P) and the free enzyme is recovered within about 2 s. In this paper, only the formation of the ES complex up to 50 ms (gray, blurred vertical line) was explored.

On the timescales employed here, the formation of later intermediates and the catalytic turnover of BlaC with CEF do not play a role (Boyd & Lunn, 1979 ▸; Hugonnet & Blanchard, 2007 ▸; Tremblay *et al.*, 2010 ▸; Olmos *et al.*, 2018 ▸). Both processes unfold over much longer timescales (Fig. 3 ▸) than the time delays examined here.

CEF binding to the active sites of BlaC is dependent on the free BlaC concentration in the crystal and the rate coefficients that describe the binding kinetics (Fig. 3 ▸). Here, the rate coefficient *k*_on_ of 3.2 *M* ^−1^ s^−1^ as estimated by Olmos and coworkers was used. The *k*_off_ rate coefficient (dashed arrow in Fig. 3 ▸) was assumed to be negligible relative to the on-rate coefficient. There is only one free parameter, the diffusion coefficient *D*, which can be inferred by matching calculated occupancies to the refined occupancies observed at Δ*t*_m_. (3) provides substrate (CEF) concentrations in each individual voxel (at each position in the crystal) at any particular time *t*. CEF binding to BlaC was calculated for each voxel by numerically integrating the rate equation with time intervals d*t*, 

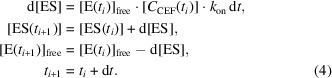

In (4), d[ES] is the change in concentration of the BlaC–CEF complex ([ES]) given the free enzyme ([E]) and free CEF ([*C*_CEF_]) concentrations at time *t*_*i*_ and the *k*_on_ rate. The free-enzyme concentration [E] decreases and that of the BlaC–CEF complex increases with each time step. (4) is repeated by increasing *t*_i_ by d*t* until *t*_*i*_ is larger than a given delay time, for example 10 ms. The substrate [*C*_CEF_] is provided everywhere by diffusion (3) and its concentration is also dependent on *t*_*i*_. The goal was to reproduce the approximate 50% BlaC–CEF occupancy in the *B* and *D* subunits (occ_obs_) which was observed in the experiment at around 5 ms. The calculated BlaC–CEF occupancy (occ_calc_) is the average of the calculated occupancies found in all voxels of the crystal. Occ_calc_ can then be compared with occ_obs_ and adjusted by varying the diffusion coefficient of CEF.

## 3. Results and discussion   

### 3.1. Formation of the enzyme–substrate complex   

The EuXFEL 564 kHz pulse structure was used to measure the binding of the large CEF substrate to BlaC at a Δ*t*_m_ of 5, 10 and 50 ms. The Δ*t*_m_ of 5 ms is about an order of magnitude faster than the earliest (30 ms) time point collected previously (Olmos *et al.*, 2018 ▸). The ∼2 µm thin, platelet-shaped BlaC microcrystals allow fast diffusion times across the thin dimension. Furthermore, diffusion is facilitated by large channels in the crystals (Olmos *et al.*, 2018 ▸). Therefore, these crystals are ideally suited for mix-and-inject investigations on fast timescales.

**Figure 4:**
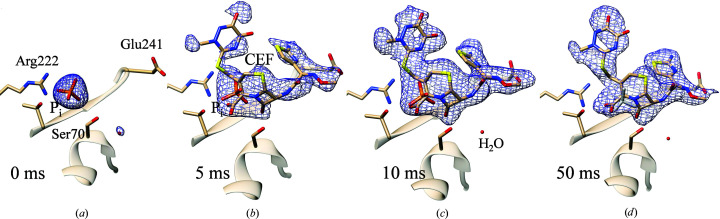
Polder difference electron density, contour level 3σ, in the active center of BlaC subunit *B*. (*a*) The CEF ligand has not yet diffused in; the phosphate (P_i_) from the crystallization buffer is dominant in the active site. (*b*) 5 ms after mixing: the phosphate is beginning to be displaced by CEF. (*c*) 10 ms after mixing: the phosphate is no longer dominant. (*d*) 50 ms after mixing: little evidence of the phosphate remains and the density only has features of the antibiotic compound. Nearby amino acids are marked in (*a*).

As observed in the previous studies at longer Δ*t*_m_, CEF only binds to BlaC subunits *B* and *D*. In Figs. 4 ▸(*b*)–4 ▸(*d*) polder difference electron-density maps (Liebschner *et al.*, 2017 ▸; DED_Polder_) are shown in the active site of subunit *B*. On early timescales (5 and 10 ms after mixing) we simultaneously observe electron densities for CEF and the close-by phosphate (P_i_) molecule. Since CEF and P_i_ occupy the same space, their presence is mutually exclusive and the electron density reflects an average over sites occupied by P_i_ and others occupied by CEF. P_i_ is also found near the CEF binding site in the un­liganded (unmixed) form [Fig. 4 ▸(*a*)]. At Δ*t*_m_ = 5 ms, the P_i_ and CEF occupancies are both approximately 50%. The available catalytic sites in subunits *B* and *D* are equally occupied either by a CEF or by a P_i_. At Δ*t*_m_ = 50 ms, the P_i_ density vanishes. Nevertheless, the P_i_ occupancy refines to 19% and that of CEF to 82% (Table 3 ▸). Here, an electron-rich compound (P_i_) is refined in conjunction with CEF, occupying equivalent spaces in different unit cells. This may result in an overestimate of the occupancy of P_i_. As there is no indication of phosphate-shaped electron density at 50 ms [Fig. 4 ▸(*d*)], we consider this to be the error margin of our occupancy refinement.

**Figure 5:**
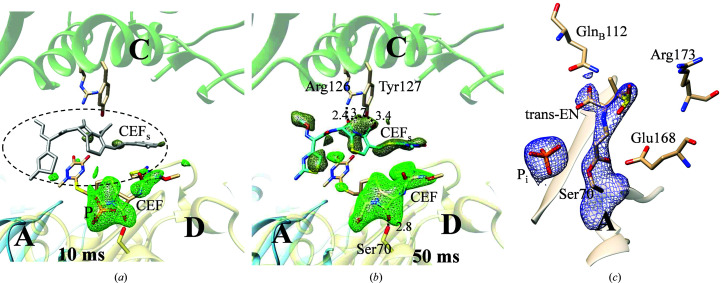
Additional ligands. (*a*) CEF in the active site and the stacking site (dotted oval) located between subunits *D* and *C*. A *DF*_o_ − *mF*_c_ OMIT difference electron-density map in the active site is shown in green and that in the stacking site in gray–green (at a 2.5σ contour level). Substantial CEF density in the active site is shown in green. There is also electron density for P_i_ present due to averaging over all unit cells in the crystal. The stacking site is not occupied (gray CEF_s_ molecule). (*b*) At 50 ms the maximum occupancy of CEF in the active center is reached. The stacking site is substantially occupied (blue CEF molecule). Important residues and distances are marked in Å. (*c*) The covalently bound *trans*-EN is present in subunit *A* of the static cryostructure of BlaC when soaked with SUB (blue; 2*F*_o_ − *F*_c_ map at a 1.5σ contour level).

**Figure 6:**
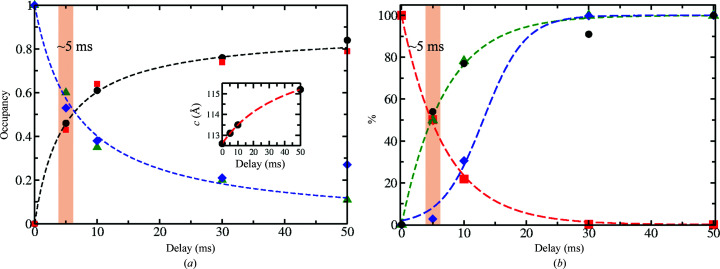
BlaC–CEF complex formation as a function of time. (*a*) Occupancies of CEF in the active site of BlaC at 5, 10, 30 and 50 ms in subunits *B* (spheres) and *D* (squares) as well as those of P_i_ (green triangles and blue diamonds) are plotted as a function of delay (the 30 ms data are from Olmos *et al.*, 2018 ▸). The data are fitted with saturation curves [equations (1) and (2), black and blue dashed lines]. The two curves intersect at around 6 ms. Inset: the corresponding change in the unit-cell axis *c*. (*b*) Concentrations (in %) as calculated from diffusion and binding [equations (1)–(5)]; green dashed line and green triangles, increase of the calculated BlaC–CEF complex concentration averaged over all voxels in the crystal; red dashed line and squares, decrease of the free enzyme (BlaC); blue dashed line and diamonds, increase of the BlaC–CEF complex in the center of the platelet-shaped crystals. For comparison, the observed (refined) occupancies of the BlaC–CEF complex (normalized to 100% at 50 ms) are also shown (black spheres).

In agreement with previous work (Olmos *et al.*, 2018 ▸), an additional CEF molecule is identified close to each active site that weakly interacts (stacks) with the CEF already bound there (Fig. 5 ▸). The stacking sites seem to be only transiently visited by CEF molecules until the active sites are fully occupied. The unit-cell parameter changes roughly follow CEF binding and P_i_ release [Fig. 6 ▸(*a*), inset; Table 1 ▸]. When sulbactam, which is about 2.5 times smaller, binds the P_i_ is not replaced and the unit-cell parameters do not change (see below and Table 1 ▸). We postulate that the displacement of the strongly negatively charged P_i_, as well as the occupancy of the stacking site, may contribute to the unit-cell changes observed when CEF is mixed in. The needle crystal form described earlier (Olmos *et al.*, 2018 ▸) does not show unit-cell changes. There, neither the P_i_ nor the stacking site is present. In our BlaC platelets the CEF occupancy can be very heterogenous, in particular at 5 ms, which should result in different unit-cell parameters near the surface and in the center, respectively. However, the Bragg reflections are not split, which is in accordance with observations by others (Ramakrishnan *et al.*, 2021 ▸; Stagno *et al.*, 2017 ▸; Kupitz *et al.*, 2014 ▸). This may be a consequence of the fully coherent illumination of the entire microcrystal volume by the XFEL radiation or may be due to the plasticity of microcrystals that even supports phase transitions and space-group changes (Ramakrishnan *et al.*, 2021 ▸).

As more CEF binds, Ser70 moves towards the P_i_ position (by about 1 Å) and the P_i_ is replaced at the same time (Table 4 ▸ *c*). Other amino acids such as Asn172 and Asp241 move closer to the CEF. We can now develop a mini-movie for the formation of an enzyme–substrate (ES) complex (Supplementary Movie S1). This movie visualizes how CEF interacts with BlaC. The initial binding phase is complete when the CEF occupancy approaches saturation. Since the aminothiazole ring and, in particular, the dioxotriazine ring of CEF stick out from the center [the β-lactam ring fused to the six-membered thiazine ring; Fig. 1 ▸(*b*)], they are more disordered and their electron densities are weaker. However, clear density features guide a structural refinement that shows that CEF binds through a succession of conformations which may be associated with distinct BlaC intermediates. The separation of these intermediates from the X-ray data is difficult since we have not collected a sufficiently large number of time delays to apply meaningful deconvolution algorithms (Schmidt *et al.*, 2003 ▸; Kostov & Moffat, 2011 ▸). The high X-ray pulse-repetition rate of the EuXFEL may make this possible since it allows the fast collection of data sets at tightly spaced delays. Given the resolution of our X-ray data (Table 1 ▸), it is challenging to make a distinction between ligand binding being supported by conformational disorder (Tzeng & Kalodimos, 2012 ▸) or by adaptation of the structure to a changing energy landscape, which would resemble an ‘induced fit’ (Changeux & Edelstein, 2011 ▸). Both scenarios (Vogt & Di Cera, 2012 ▸) would most likely result in the same (or a very similar) crystallographic signal. We hypothesize that both mechanisms are involved to some degree, which might be unraveled by single-particle experiments, as recently demonstrated for an unrelated biological system (Dashti *et al.*, 2020 ▸). However, the structures of BlaC as well as of CEF change [Table 4 ▸(*c*), Supplementary Movie S1], which might be interpreted as the signature of an induced fit after the initial binding event.

Formation of the ES complex is most important since it triggers the enzymatic cycle. Hence, it determines the time resolution of the MISC method. The ES complex consists of CEF noncovalently bound in the active site of BlaC (Fig. 4 ▸). CEF is delivered by diffusion into the crystals. The crystals must be small enough to enable short enough diffusion times so that the binding kinetics can be observed. However, MISC does not measure the free substrate concentration in the crystals, and therefore diffusion is rather observed indirectly through the increase in the occupancies of well ordered substrate molecules in the active centers of BlaC. When the diffusion times are very short, occupancies may accumulate on a timescale longer than the diffusion time, as they are governed by the binding kinetics. The formation of the ES complex, and therefore the time resolution of the MISC method, is therefore not only dependent on the ligand concentration delivered by diffusion but also on the magnitude of the rate coefficients that characterize the kinetic mechanism.

### 3.2. Inhibitor binding   

**Figure 7:**
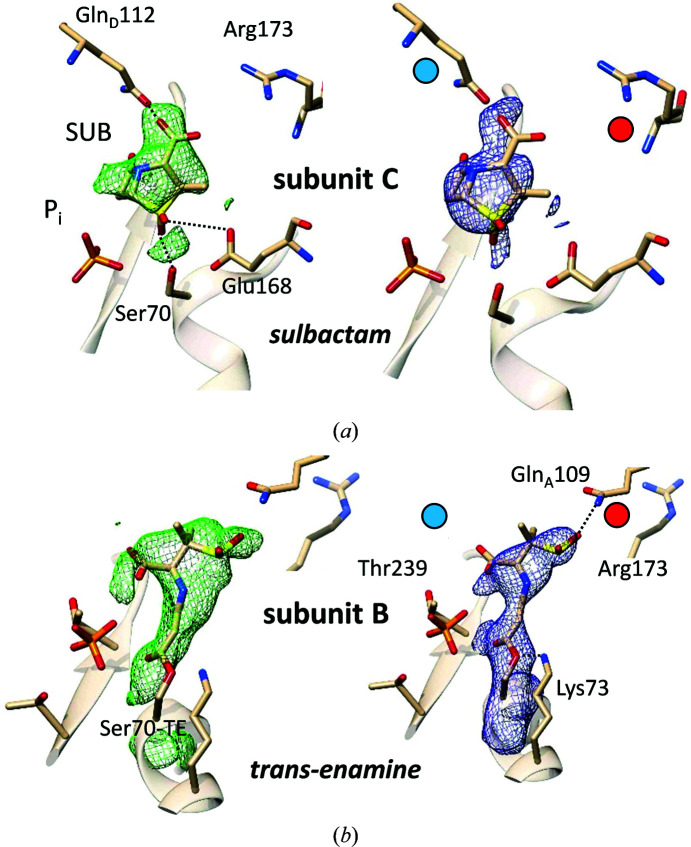
BlaC–SUB complexes at Δ*t*_m_ = 66 ms. (*a*) Active site in subunit *C* with noncovalently bound intact sulbactam; left side, DED_iso_ map (contour: 3σ); right side, 2*mF*_o_ − *DF*_c_ map (contour: 1.7σ) after refinement. Close-by amino acids and the phosphate (P_i_) are marked. (*b*) Active site in subunit *B* with *trans*-enamine bound to Ser70; left side, DED_iso_ map (contour: 3σ); right side, 2*mF*_o_ − *DF*_c_ map (contour: 1.7σ) after refinement. Red and blue dots show important differences between the subunits. Gln112 from the adjacent subunit is not located close by and Arg173 is extended in subunit *B*, leaving subunit *B* more accessible to ligands and substrate.

The structure of the BlaC–SUB complex was probed at Δ*t*_m_ = 66 ms. Strong positive DED_iso_ shows SUB binding to all four subunits of BlaC, which is in stark contrast to CEF, which only binds to subunits *B* and *D*. The absence of negative DED_iso_ at the P_i_ position [Figs. 7 ▸(*a*) and 7 ▸(*b*)] shows that the P_i_ does not move and stays in the active site. At the time delay of 66 ms sulbactam binding to BlaC is heterogeneous. In subunits *B* and *D* the DED_iso_ is elongated and stretches outwards from Ser70 [Fig. 7 ▸(*b*)]. In subunits *A* and *C* the DED_iso_ is pillow-like and is more distant from Ser70 [Fig. 7 ▸(*a*)]. The elongated DED_iso_ in subunits *B* and *D* [Fig. 7 ▸(*b*)] can be explained by a covalently bound *trans*-EN as a result of the reaction of the sulbactam with the catalytic Ser70. The diffusion time is fast enough that 66 ms after mixing all *B* and *D* subunits contain *trans*-EN, the position of which is stabilized by a network of BlaC residues [Fig. 7 ▸ and Table 4 ▸(*b*)]. This is quite unexpected, as it was suggested that it would take minutes for the enamine to form after binding of SUB to BlaC (Totir *et al.*, 2007 ▸; Cheng *et al.*, 2020 ▸; Tassoni *et al.*, 2019 ▸). In subunits *B* and *D* Arg173 displays a stretched, open conformation, allowing the SUB to orient correctly towards Ser70 and to react swiftly to the *trans*-EN, which then irreversibly inhibits BlaC (Tassoni *et al.*, 2019 ▸). The nearby P_i_, which is displaced when the much larger ligand CEF is present, stays in place in all subunits and is likely to add to the stability of both complexes.

The pillow-like DED_iso_ in a region more distant from Ser70 in subunits *A* and *C* [Fig. 7 ▸(*a*)] can be explained by an intact sulbactam molecule that is noncovalently bound to the active site. The SUB is oriented so that the ring sulfur dioxide points towards Ser70, with the β-lactam ring pointing away from Ser70. We hypothesize that this ‘upside-down’ orientation is enforced by Arg173 and Gln112 [Fig. 7 ▸(*a*) and Table 4 ▸(*a*)], where Gln112 protrudes deep into the active sites from the adjacent, noncrystallographically related subunits [Fig. 7 ▸(*a*)]. As the noncovalently bound SUB is oriented incorrectly, Ser70 cannot attack and open the β-lactam ring within the Δ*t*_m_ of 66 ms. However, the static (cryo) X-ray structure of this complex [Fig. 5 ▸(*c*)] shows that SUB indeed also reacts to the *trans*-EN in subunits *A* and *C*. Accordingly, the BlaC–SUB structure is an interesting intermediate on the catalytic pathway from SUB to *trans*-EN. The detection of this intermediate would be difficult if not impossible without the MISC experiment.

### 3.3. Diffusion of substrate in BlaC microcrystals   

**Figure 8:**
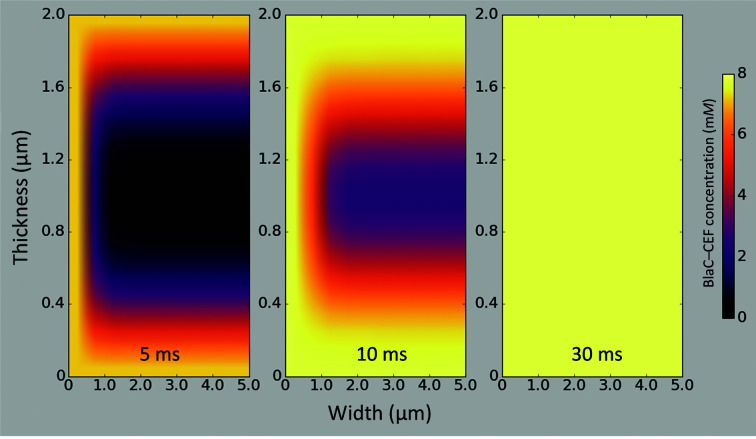
Concentrations of the BlaC–CEF complex in 10 × 10 × 2 µm platelet-shaped crystals (*a*) 5 ms, (*b*) 10 ms and (*c*) 30 ms after mixing with 200 m*M* ceftriaxone (150 m*M* final concentration assumed). The concentrations are shown in different colors (see the scale bar on the right) in central cross sections through half the width of the crystals. The drawings are not to scale, since the sections displayed are 5 µm horizontally (width) and 2 µm vertically (thickness). The enlargement along the short 2 µm axis allows the display of the nuanced occupancy differences.

**Figure 9:**
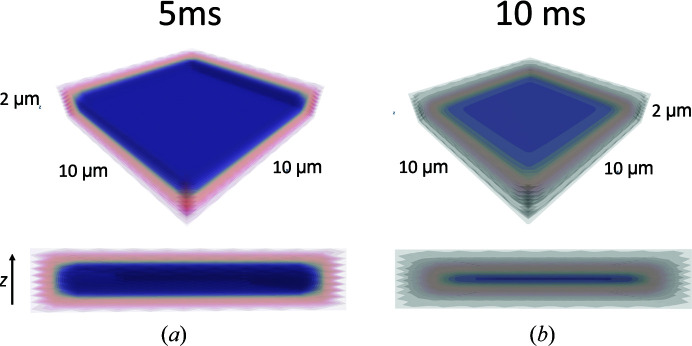
3D representation of CEF occupancy values in the BlaC catalytic cleft 5 ms (*a*) and 10 ms (*b*) after mixing in a typical BlaC microcrystal platelet. Dark blue colors denote low occupancies and lighter hues denote high occupancies.

With *D* = 2.3 × 10^−6^ cm^2^ s^−1^ for CEF in water [Table 5 ▸(*b*)], the diffusion time into the center of a 10 × 10 × 2 µm crystal volume consisting of water is 1.6 ms. This means that at 5 ms the concentration of CEF molecules would be 96% of the outside concentration (about 144 m*M*), which is about 20 times higher than the stoichiometric concentration [Table 5 ▸(*a*)]. After integration of the rate equation, the average occupancy would be 99%, which is essentially saturation. This result does not reflect the crystallographically observed occupancy at 5 ms. When decreasing the diffusion coefficient to 2.0 × 10^−7^ cm^2^ s^−1^, the diffusion time into the crystal center increases to 19 ms [see Fig. 6 ▸(*b*) and Table 5 ▸(*b*)]. From this, the decrease of the free BlaC enzyme concentration, the increase of the free CEF concentration and the increase of the BlaC–CEF complex concentration were calculated as explained in Section 2.5. The results are shown in Fig. 6 ▸(*b*). The BlaC–CEF concentration in the center of the crystal lags behind [blue diamonds in Fig. 6 ▸(*b*)] since CEF requires additional time to reach the center and to bind to BlaC. The resulting sigmoidal-shaped response was fitted by a logistic function,

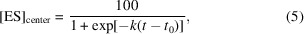

where *t*_0_ = 13.1 ms denotes the characteristic time when the binding reaction accelerates in the crystal center. With *k* = 0.3 ms^−1^ a reasonable steepness of (5) was achieved [Fig. 6 ▸(*b*), blue dashed line]. The *t*_0_ roughly coincides with the diffusion time. Averaged over the entire crystal volume, ∼50% BlaC–CEF occupancy is reached at 5 ms, which is equal to the occupancy determined experimentally at Δ*t*_m_ = 5 ms [compare the green dashed line in Fig. 6 ▸(*b*) with Fig. 6 ▸(*a*)]. Fig. 8 ▸ shows a heatmap that plots BlaC–CEF occupancies through the center of half a BlaC crystal (see also Fig. 9 ▸ for a 3D representation). The concentrations of the BlaC–CEF complex are depicted with various colors (see the scale bar on the right). At the edge of the crystal, almost all active BlaC subunits (*B* and *D*) are already bound to CEF at 5 ms. In the center the BlaC–CEF complex concentration is 0.21 m*M* (2.7% of the total concentration of *B* and *D* subunits in the crystal), although the CEF concentration delivered by diffusion is already 35 m*M* (which is the mentioned 23% of the outside CEF concentration but is 5.5 times the stoichiometric concentration). The situation changes completely at 30 ms, where almost 100% occupancy is reached everywhere in the crystal, which is in accordance with earlier results (Olmos *et al.*, 2018 ▸) and with the occupancy at Δ*t*_m_ = 50 ms reported here (see also Supplementary Movie S2). As also discussed earlier, the variation of occupancy across microcrystals at faster mix-and-inject delays does not affect the enzyme kinetics, as the nucleophilic attack of Ser70 of BlaC on the β-lactam ring happens long after the crystals establish full CEF occupancy. It needs to be pointed out here that the simple model of CEF diffusion into BlaC microcrystals and the binding of CEF to BlaC molecules can be augmented by taking into account, for example, the exclusion volume occupied by reacting and nonreacting BlaC subunits (Geremia *et al.*, 2006 ▸), by the mentioned pointwise depletion of the free CEF concentration in each voxel by binding to BlaC active centers, or the diffusion of substrate directly through protein molecules facilitated by protein dynamics. Since the exact mechanism of diffusion through protein crystals (Geremia *et al.*, 2006 ▸) is difficult to determine, the unknown parameters are tied up in the effective diffusion coefficient (*D*_eff_) determined here.

### 3.4. Reaction initiation by diffusion   

CEF diffusion is about a factor of 12 slower in the BlaC crystals than in water, with a *D*_eff_ of about ∼2 × 10^−7^ cm^2^ s^−1^ [Table 5 ▸(*b*)]. This slowdown is in agreement with findings that were previously obtained from simulations on substrate diffusion in enzyme crystals (Geremia *et al.*, 2006 ▸). Estimates of enzyme–ligand occupancies can now be directly deduced from time-resolved X-ray crystallography everywhere in a crystal after mixing. Not surprisingly, at 5 ms the occupancy is high (>90%) only near the crystal surface [Fig. 8 ▸(*a*)], where sufficient substrate is present to promote ES formation at a high rate. In the center of the crystals the ES complex concentration is initially small [Table 5 ▸(*c*), Figs. 8 ▸(*a*) and 9 ▸(*a*)]. The binding rate is not sufficiently high to generate significant occupancy. After Δ*t*_m_ = 10 ms the binding rate increases, until at 30 ms full occupancy of the BlaC–CEF complex is reached everywhere (Olmos *et al.*, 2018 ▸) [Fig. 6 ▸(*b*), green dashed line, Table 5 ▸(*c*)]. With the rapid diffusion of CEF into small BlaC crystals, we are now able to quantify variations of substrate, enzyme and ES concentrations across the enzyme crystal volume at any time [Table 5 ▸(*c*), Figs. 8 ▸ and 9 ▸]. The remarkable speed of ES accumulation shows that the mix-and-inject technique can be used to characterize enzymes with turnover times much faster than that of BlaC. The direct observation of the important initial ligand- and substrate-binding phase in biomedically relevant enzymes is possible at the EuXFEL.

Since the ES complex (here the BlaC–CEF complex) triggers the enzymatic cycle, accurate kinetics can be extracted to the point where the time required to accumulate sufficient ES complex approaches the lifetime of the next intermediate in the catalytic cycle (Schmidt, 2013 ▸). This finding holds for any other technique (Šrajer & Schmidt, 2017 ▸) which aims to trigger enzymatic reactions, even in noncrystalline samples. Not only is it required to bring sufficient substrate into the vicinity of the enzyme, but the binding kinetics also need to be taken into account. With microcrystals below a certain crystal size, the binding of substrate, and not the diffusion of substrate into the crystal volume, may become rate-limiting. As a consequence, for BlaC crystals of a size of about 1 µm the speed of ES complex formation is not substantially different from that in solution. The *D*_eff_ determined here suggests that accurate measurements of the substrate-binding kinetics would not be possible with significantly larger crystals. Enzymes with turnover times faster than that of BlaC will usually also display faster substrate-binding kinetics with larger *k*_on_ rate coefficients. In such cases, the crystal sizes (and their size distributions) or perhaps the temperature must be adjusted appropriately to ensure that the diffusion times can catch up with the substrate-binding rates.

Diffusion is an effective way to initiate reactions. Given a sufficient substrate concentration, and appropriately small crystals, all of the crystal volume is already infused with a multiple of the stoichiometric substrate concentration after a few milliseconds [Table 5 ▸(*c*)]. This is very important for fast reaction initiation since the rate (speed) of enzyme–substrate complex formation, and therefore the time resolution of the method, depends decisively, and primarily, on the concentration of the substrate, and of course also on the free enzyme concentration and the kinetic rate coefficients. Others (Mehrabi, Schulz, Dsouza *et al.*, 2019 ▸) have reported significant substrate occupancy in the active site only 30 ms after the activation of a caged substrate that is even located close by. This slow occupancy increase may be a result of (sub-)stoichiometric substrate concentrations in the unit cell, which strengthens our point of view. BlaC is not a fast enzyme. Apart from the possibility of investigating the initial substrate-binding phase(s) potentially on submillisecond timescales, the benefits of XFEL-based mix-and-inject approaches may come to light once faster enzymes with turnover times of <50 ms are investigated. These experiments require small crystals. Exploration of how to investigate these small crystals using either XFELs or synchrotrons, perhaps after upgrade to an advanced accelerator lattice (Eriksson, 2016 ▸; Wanzenberg *et al.*, 2019 ▸), remains to be performed.

## 4. Outlook   

In order to further investigate CEF and SUB binding and their reactions with Ser70, a time series should be collected that consists of data sets at multiple Δ*t*_m_ values that span from a few milliseconds to seconds. To achieve this, the EuXFEL pulse structure must be exploited most efficiently. Every X-ray pulse in all pulse trains provides observations of the same time delay, and our experiments took maximum advantage of the high pulse rate (Fig. 2 ▸, inset). This is in contrast to optical pump–X-ray probe experiments, which require appropriate waiting times between laser excitations to guarantee that the laser-excited volume exits the X-ray interaction region, so that multiple laser activations can be avoided (Pandey *et al.*, 2020 ▸). We showed that diffraction data sufficient for good-quality structure determination can be collected in about half an hour, as demonstrated for the 50 ms CEF time point. This time can be reduced substantially by limiting the number of diffraction patterns per data set (around 25 000 is appropriate for this space group; Olmos *et al.*, 2018 ▸) and by optimizing the crystal density that flows through the mixing device. High crystal density will lead to higher hit rates, but might also cause frequent interruptions caused by injector clogging. In our experiments, a fine balance between crystal size and crystal density was found so that the mix-and-inject experiments with CEF and SUB could be completed successfully with acceptable hit rates (Table 1 ▸) given the high X-ray pulse-repetition rate at the EuXFEL. Previous experiments have shown that the collection of sufficient patterns for structure determination should be possible in less than 20 min at the detector-limited repetition rate of the EuXFEL (Yefanov *et al.*, 2019 ▸; Pandey *et al.*, 2020 ▸). This provides the tantalizing possibility of directly characterizing kinetic processes in biomolecules from single-digit millisecond to longer timescales within relatively short experimental times. The kinetics can rapidly change when environmental conditions are varied. It may be possible, for example, to control the temperature in the mixing injector delay line to determine barriers of activation from the resulting X-ray data (Schmidt *et al.*, 2013 ▸). The full analysis of such a multidimensional data set requires the development and deployment of user-friendly classification algorithms to separate mixtures into their pure components (Schmidt *et al.*, 2003 ▸) and to derive kinetics and energetics (Schmidt *et al.*, 2013 ▸) consistent with the electron-density maps and the structures of intermediate states along the reaction pathway.

## 5. Summary   

Our experiments permitted a real-time view into the active sites of an enzyme during substrate binding. They facilitate more mix-and-inject experiments at the EuXFEL with unprecedented data-collection rates, allowing more structures to be determined per allocated experimental time. This capability will become an important tool for biomedically relevant research in years to come.

## Supplementary Material

PDB reference: BlaC, unmixed, 7k8l

PDB reference: mixed with ceftriaxone, 5 ms, 7k8e

PDB reference: 10 ms, 7k8f

PDB reference: 50 ms, 7k8h

PDB reference: mixed with sulbactam, 66 ms, 7k8k

## Figures and Tables

**Figure 10:**
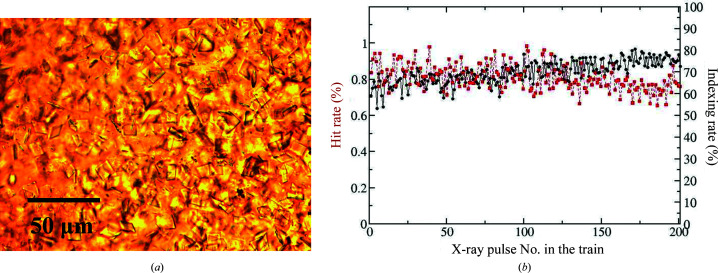
Crystals, hit and indexing rates. (*a*) Microscopic image of the platelet crystal form of BlaC and (*b*) exemplary hit (red squares) and indexing (black spheres) rates from BlaC/CEF mixing as a function of the pulse ID in the train. Both rates are stable across the entire pulse train.

**Figure 11:**
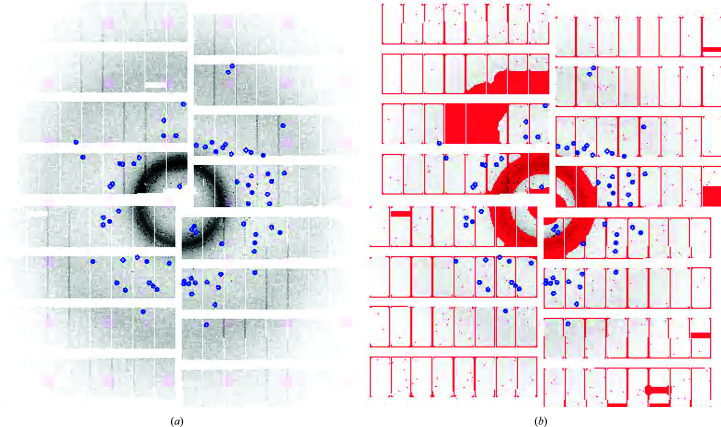
(*a*) Example of a diffraction pattern collected by the AGIPD. In (*b*) a mask (in red) is superposed that covers the strong rings at low resolution and inactive detector areas.

**Table 1:** Data-collection statistics [30 ms data from Olmos *et al.* (2018 ▸)]

	Water (reference)	5 ms CEF	10 ms CEF	50 ms CEF	66 ms SUB	30 ms CEF (LCLS)
Temperature (K)	293	293	293	293	293	293
Space group	*P*2_1_	*P*2_1_	*P*2_1_	*P*2_1_	*P*2_1_	*P*2_1_
EuXFEL train pulse rate (kHz)	564	564	564	564	564	564
*a*, *b*, *c* (Å)	80.9, 99.5, 112.6	80.6, 98.7, 113.1	80.6, 98.5, 113.5	80.4, 98.2, 115.2	81.0, 99.5, 112.6	78.7, 96.8, 112.6
α, β, γ (°)	90, 108.4, 90	90, 108.6, 90	90, 108.8, 90	90, 110.0, 90	90, 108.4, 90	90, 109.7, 90
Resolution (Å)	2.8	2.4	2.6	2.6	2.7	2.7
Hits	51980	110698	85775	85914	35886	35065
Indexed patterns	31812	105495	52323	36256	25013	24397
Hit/indexing rate (%)	2.98/61.2	0.65/95.3	1.33/61.0	2.26/42.2	0.78/69.7	3.87/69.5
Observed reflections	31572191	114717921	49576617	38055135	21034155	14588166
Unique reflections	41870	65232	51595	50760	45344	40340
Multiplicity	754 (236)	1758 (1246)	966.3 (580.4)	749.7 (449.4)	463.8 (307.4)	526 (142)
Completeness (%)	100 (100)	100 (100)	100 (100)	100 (100)	100 (100)	100 (100)
*R*_split_ (%)	20.6 (988)	15.6 (303.7)	17.8 (334)	20.9 (198.1)	21.4 (459.2)	14.2 (121.1)
CC_1/2_ (%)	96.5 (22.9)	99.2 (26.9)	99.6 (58.4)	99.5 (58.4)	96.9 (20.5)	98.6 (34.5)

**Table 2:** Parameters for the mix-and-inject experiments Concentrations of ceftriaxone (CEF) and sulbactam (SUB) are shown as were flowed through the outer capillary line of the mixing injectors. Time delays are achieved after mixing in a constriction as per Calvey *et al.* (2019 ▸).

	Water	SUB	CEF	CEF	CEF
Δ*t*_m_ (ms)	(10)	66	5	10	50
Ligand concentration (m*M*)	—	100	200	200	200
Ligand buffer	—	0.8 *M* AP pH 4.6	0.8 *M* AP pH 4.6	0.8 *M* AP pH 4.6	0.8 *M* AP pH 4.6
Ligand flow (µl min^−1^)	74.5	54.5	76.7	74.5	71.8
Crystal flow (µl min^−1^)	5.5	11.6	3.3	5.5	8.2
Mixing injector capillary internal diameter (µm)	50	75	50	50	75
Constriction length (mm)	17.8	36.1	9.3	17.8	36.1
Timing uncertainty (ms)	—	9.3	1.8	3.0	10.4
Experimental time to collect the data set (min)	50	56	138	250	32

**Table 3:** Refinement statistics

	Water	5 ms CEF	10 ms CEF	50 ms CEF	66 ms SUB	30 ms CEF (Olmos *et al.*, 2018 ▸, revisited)
Refinement program	*Phenix*	*Phenix*	*Phenix*	*Phenix*	*REFMAC*	*Phenix*
Resolution	2.8	2.4	2.6	2.6	2.7	2.75
Reflections used	36804	52163	43274	45264	36434	40951
*R*_cryst_/*R*_free_	0.21/0.27	0.24/0.25	0.22/0.26	0.22/0.27	0.21/0.29	0.22/0.26
Occupancy (CEF/phosphate) (%)	—	*B*, 46/60; *D*, 43/53	*B*, 61/35; *D*, 64/38	*B*, 84/11; *D*, 79/27	100, not refined	*B*, 76/20; *D*, 74/21
〈*B*〉_CEF_ (*B*/*D*) (Å^2^)	—	51/48	55/58	53/50	—	70/67
R.m.s.d., bond lengths (Å^2^)	0.009	0.003	0.003	0.003	0.007	0.003
R.m.s.d., bond angles (°)	1.09	1.07	1.03	1.34	1.67	1.10
H_2_O	129	246	251	247	201	146

**Table:** (*a*) Distances in (Å) in subunits *A* and *C* to the sulbactam (0RN).

	Subunit *A*	Subunit *C*
Ser70 OG to P_i_ O	3.2 (h)	2.6 (h)
Ser70 OG to 0RN OAF	3.1 (h)	3.2 (h)
Gln112 OE1 to 0RN OX	Gln from *B*, 2.8 (h)	Gln from *D*, 3.3 (w)
Asn172 ND2 to 0RN OAF	3.2 (h)	3.1 (h)
Glu168 O2 to 0RN OAF	3.0 (h)	2.8 (h)
Arg173 NH1 to 0RN O	2.5 (s)	3.9 (w)
Thr239 O to 0RN OAO	3.9 (w)	2.9 (h)

**Table:** (*b*) Distances in (Å) in subunits *B* and *D* to the Ser70 *trans*-enamine (TSS).

	Subunit *B*	Subunit *D*
Lys73 NZ to TSS O13	2.8 (h)	3.0 (h)
Gln109 OE1 to TSS O12	Gln from *A*, 2.7 (h)	Gln from *C*, 2.9 (h)
Thr239 O to TSS 08	2.9 (h)	2.9 (h)
Asp241 OD2 to TSS O11	6.4	4.1

**Table:** (*c*) Distances in (Å) during ceftriaxone binding (CEF is only bound to subunits *B* and *D*).

	Subunit *B*				Subunit *D*			
	0 ms	5 ms	10 ms	50 ms	0 ms	5 ms	10 ms	50 ms
Ser70 OG to H_2_O	3.1 (h)	2.8 (h)	3.3 (w)	2.8 (h)	3.5 (w)	2.4† (s)	3.1 (h)	3.2 (h)
Ser70 OG to P_i_ O4	3.6 (w)	3.5 (w)	3.4 (w)	2.6 (s)	2.7 (h)	3.7 (w)	3.9 (w)	2.7 (h)
Ser70 OG to CEF O	na‡	3.1 (h)	2.9 (h)	2.9 (h)	na	2.9 (h)	3.0 (h)	2.8 (h)
Ser128 OG to CEF OAD	na	2.4 (s)	2.4 (s)	2.5 (s)	na	2.3 (s)	2.4 (s)	2.6 (h)
Asn172 ND2 to CEF OAR	na	2.7 (h)	3.1 (h)	3.2 (h)	na	2.8 (h)	3.0 (h)	3.1 (h)
Thr237 OG1 to CEF OA1	na	2.7 (h)	3.1 (h)	3.0 (h)	na	2.7 (h)	2.6 (h)	3.1 (h)
Thr239 OG2 to CEF OA1	na	3.3 (w)	3.0 (h)	3.0 (h)	na	3.4 (w)	3.4 (w)	3.1 (h)
Asp241 OD1 to CEF NAC	na	**4.7**	**4.3**	**3.2** (h)	**na**	**4.4**	**4.2**	**3.6** (w)

† Weak OMIT difference electron density.

‡ Not applicable.

**Table:** (*a*) Parameters for the binding of CEF to BlaC [see also Fig. 3 ▸ and equation (4)]. The concentration *E*_0_ of all subunits in the BlaC platelet crystal form is 15.5 m*M*. Only subunits *B* and *D* bind substrate. *C*_0,CEF_ is the mixed-in substrate concentration [see equation (3)].

*E*_0_ (m*M*)	*C*_0,CEF_ (m*M*)	*k*_on_ (*M* ^−1^ s^−1^)
7.8	150	3.2

**Table:** (*b*) Parameters in equations (1)–(5) that were fitted to the respective refined occupancy values of CEF and P_i_. The comparison of observed and calculated occupancies allows the determination of a diffusion coefficient *D*_eff_.

BlaC–CEF increase, (1)		P_i_ decrease, (2)	BlaC–CEF in the crystal center, (5)			
Observed†		Observed†	Sigmoidal increase‡		*D* (cm^2^ s^−1^) for CEF, (3) and (4)	
*C*_S,CEF_	τ_1/2_ (ms)	τ_1/2_ (ms)	*k* (ms^−1^)	*t*_0_ (ms)	Water	*D*_eff_ §
88%	4.6	6.7	0.3	13.1	2.3 × 10^−6^	2 × 10^−7^

**Table:** (*c*) Calculated occupancies which could be determined with the help of *D*_eff_. The free CEF, free BlaC ([E_free_]) and BlaC–CEF complex ([ES]) concentrations were averaged (angle brackets) over all voxels in the crystal. [ES_center_] is the BlaC–CEF complex concentration in the center of the microcrystal platelets. Values in parentheses either denote the inside CEF concentration in terms of the percentage of the outside CEF concentration or represent the occupancy values of the relevant species.

Δ*t*_m_ (ms)	〈[CEF]〉 (m*M*)	〈[E_free_]〉 (m*M*)	〈[ES]〉 (m*M*)	[ES_center_] (m*M*)
5	79.4 (53%)	3.9 (50%)	3.9 (50%)	0.2 (2.7%)
10	98.4 (66%)	1.7 (21%)	6.1 (79%)	2.4 (30%)
30	133.3 (89%)	0.0 (0.1%)	7.8 (99.9%)	7.7 (99%)
50	144.4 (96%)	0.0 (0%)	7.8 (100%)	7.8 (100%)

† From fitting saturation curves to refined occupancy values. *C*_S,CEF_ is the saturation concentration of CEF; τ_1/2_ are characteristic times where 50% of the final concentrations of CEF and phosphate are reached, respectively.

‡ Parameters of the logistics function (5) fitted to occupancies determined in the centers of the BlaC platelets.

§ *D*_eff_ was obtained by matching the calculated and observed CEF binding kinetics.

## Appendix A Microcrystals and data collection

Fig. 10 ▸(*a*) shows a microscopic image of the BlaC platelets used for the experiments. They appear to be uniform in size and shape. In Fig. 10 ▸(*b*) the hit and indexing rates achieved with these crystals in our MISC experiments are displayed across all of the pulses in the pulse train of the EuXFEL. A decay in the hit rate following pulse 1 is not observed. This is an indication that the jet is fast enough that a fresh jet volume is intercepted by the following X-ray pulses (Wiedorn, Oberthür *et al.*, 2018 ▸; Yefanov *et al.*, 2019 ▸; Pandey *et al.*, 2020 ▸).

## Appendix B Diffraction patterns and masks

The ability to mask out detector pixels and other parts of the diffraction pattern (Fig. 11 ▸) is important for the successful identification of DP-containing Bragg reflections (hits) from the stream of detector images (Wiedorn, Oberthür *et al.*, 2018 ▸; Carrillo *et al.*, 2021 ▸). Only peaks [blue squares in Figs. 11 ▸(*a*) and 11 ▸(*b*)] outside the mask are found. In the hit-finder *Cheetah* (Barty *et al.*, 2014 ▸) a convenient masking tool is available that facilitates the exclusion of any part of the detector image. The central ‘hole’ and the gaps (except for the tile edges) do not need to be masked since the detector geometry is known.

## References

[bb2] Allahgholi, A., Becker, J., Delfs, A., Dinapoli, R., Goettlicher, P., Greiffenberg, D., Henrich, B., Hirsemann, H., Kuhn, M., Klanner, R., Klyuev, A., Krueger, H., Lange, S., Laurus, T., Marras, A., Mezza, D., Mozzanica, A., Niemann, M., Poehlsen, J., Schwandt, J., Sheviakov, I., Shi, X., Smoljanin, S., Steffen, L., Sztuk-Dambietz, J., Trunk, U., Xia, Q., Zeribi, M., Zhang, J., Zimmer, M., Schmitt, B. & Graafsma, H. (2019). *J. Synchrotron Rad.* **26**, 74–82. 10.1107/S1600577518016077 PMC6337892 30655470

[bb3] Barends, T. R. M., Foucar, L., Ardevol, A., Nass, K., Aquila, A., Botha, S., Doak, R. B., Falahati, K., Hartmann, E., Hilpert, M., Heinz, M., Hoffmann, M. C., Kofinger, J., Koglin, J. E., Kovacsova, G., Liang, M., Milathianaki, D., Lemke, H. T., Reinstein, J., Roome, C. M., Shoeman, R. L., Williams, G. J., Burghardt, I., Hummer, G., Boutet, S. & Schlichting, I. (2015). *Science*, **350**, 445–450. 10.1126/science.aac5492 26359336

[bb4] Barty, A., Kirian, R. A., Maia, F. R. N. C., Hantke, M., Yoon, C. H., White, T. A. & Chapman, H. (2014). *J. Appl. Cryst.* **47**, 1118–1131. 10.1107/S1600576714007626 PMC4038800 24904246

[bb5] Berman, H. M., Battistuz, T., Bhat, T. N., Bluhm, W. F., Bourne, P. E., Burkhardt, K., Feng, Z., Gilliland, G. L., Iype, L., Jain, S., Fagan, P., Marvin, J., Padilla, D., Ravichandran, V., Schneider, B., Thanki, N., Weissig, H., Westbrook, J. D. & Zardecki, C. (2002). *Acta Cryst.* D**58**, 899–907. 10.1107/s0907444902003451 12037327

[bb6] Boutet, S., Lomb, L., Williams, G. J., Barends, T. R., Aquila, A., Doak, R. B., Weierstall, U., DePonte, D. P., Steinbrener, J., Shoeman, R. L., Messerschmidt, M., Barty, A., White, T. A., Kassemeyer, S., Kirian, R. A., Seibert, M. M., Montanez, P. A., Kenney, C., Herbst, R., Hart, P., Pines, J., Haller, G., Gruner, S. M., Philipp, H. T., Tate, M. W., Hromalik, M., Koerner, L. J., van Bakel, N., Morse, J., Ghonsalves, W., Arnlund, D., Bogan, M. J., Caleman, C., Fromme, R., Hampton, C. Y., Hunter, M. S., Johansson, L. C., Katona, G., Kupitz, C., Liang, M., Martin, A. V., Nass, K., Redecke, L., Stellato, F., Timneanu, N., Wang, D., Zatsepin, N. A., Schafer, D., Defever, J., Neutze, R., Fromme, P., Spence, J. C. H., Chapman, H. N. & Schlichting, I. (2012). *Science*, **337**, 362–364.

[bb7] Boyd, D. B. & Lunn, W. H. (1979). *J. Med. Chem.* **22**, 778–784. 10.1021/jm00193a006 448675

[bb8] Calvey, G. D., Katz, A. M. & Pollack, L. (2019). *Anal. Chem.* **91**, 7139–7144. 10.1021/acs.analchem.9b00311 31060352

[bb9] Carrillo, M., Pandey, S., Sanchez, J., Noda, M., Poudyal, I., Aldama, L., Malla, T. N., Claesson, E., Wahlgren, W. Y., Feliz, D., Šrajer, V., Maj, M., Castillon, L., Iwata, S., Nango, E., Tanaka, R., Tanaka, T., Fangjia, L., Tono, K., Owada, S., Westenhoff, S., Stojković, E. A. & Schmidt, M. (2021). *Structure*, **29**, 743–754. 10.1016/j.str.2021.03.004 PMC8405169 33756101

[bb10] Carslaw, H. S. & Jaeger, J. C. (1959). *Conduction Heat in Solids*, 2nd ed. Oxford: Clarendon Press.

[bb11] Cassini, A., Högberg, L. D., Plachouras, D., Quattrocchi, A., Hoxha, A., Simonsen, G. S., Colomb-Cotinat, M., Kretzschmar, M. E., Devleesschauwer, B., Cecchini, M., Ouakrim, D. A., Oliveira, T. C., Struelens, M. J., Suetens, C., Monnet, D. L., Strauss, R., Mertens, K., Struyf, T., Catry, B., Latour, K., Ivanov, I. N., Dobreva, E. G., Tambic-Andraševic, A., Soprek, S., Budimir, A., Paphitou, N., Žemlicková, H., Schytte Olsen, S., Wolff Sönksen, U., Märtin, P., Ivanova, M., Lyytikäinen, O., Jalava, J., Coignard, B., Eckmanns, T., Abu Sin, M., Haller, S., Daikos, G. L., Gikas, A., Tsiodras, S., Kontopidou, F., Tóth, Á., Hajdu, Á., Guólaugsson, Ó., Kristinsson, K. G., Murchan, S., Burns, K., Pezzotti, P., Gagliotti, C., Dumpis, U., Liuimiene, A., Perrin, M., Borg, M. A., de Greeff, S. C., Monen, J. C., Koek, M. B., Elstrøm, P., Zabicka, D., Deptula, A., Hryniewicz, W., Caniça, M., Nogueira, P. J., Fernandes, P. A., Manageiro, V., Popescu, G. A., Serban, R. I., Schréterová, E., Litvová, S., Štefkovicová, M., Kolman, J., Klavs, I., Korošec, A., Aracil, B., Asensio, A., Pérez-Vázquez, M., Billström, H., Larsson, S., Reilly, J. S., Johnson, A. & Hopkins, S. (2019). *Lancet Infect. Dis.* **19**, 56–66.

[bb12] Changeux, J.-P. & Edelstein, S. (2011). *F1000 Biol. Rep.* **3**, 19. 10.3410/B3-19 PMC3169905 21941598

[bb13] Chapman, H. N., Fromme, P., Barty, A., White, T. A., Kirian, R. A., Aquila, A., Hunter, M. S., Schulz, J., DePonte, D. P., Weierstall, U., Doak, R. B., Maia, F. R. N. C., Martin, A. V., Schlichting, I., Lomb, L., Coppola, N., Shoeman, R. L., Epp, S. W., Hartmann, R., Rolles, D., Rudenko, A., Foucar, L., Kimmel, N., Weidenspointner, G., Holl, P., Liang, M., Barthelmess, M., Caleman, C., Boutet, S., Bogan, M. J., Krzywinski, J., Bostedt, C., Bajt, S., Gumprecht, L., Rudek, B., Erk, B., Schmidt, C., Hömke, A., Reich, C., Pietschner, D., Strüder, L., Hauser, G., Gorke, H., Ullrich, J., Herrmann, S., Schaller, G., Schopper, F., Soltau, H., Kühnel, K.-U., Messer­schmidt, M., Bozek, J. D., Hau-Riege, S. P., Frank, M., Hampton, C. Y., Sierra, R. G., Starodub, D., Williams, G. J., Hajdu, J., Timneanu, N., Seibert, M. M., Andreasson, J., Rocker, A., Jönsson, O., Svenda, M., Stern, S., Nass, K., Andritschke, R., Schröter, C.-D., Krasniqi, F., Bott, M., Schmidt, K. E., Wang, X., Grotjohann, I., Holton, J. M., Barends, T. R. M., Neutze, R., Marchesini, S., Fromme, R., Schorb, S., Rupp, D., Adolph, M., Gorkhover, T., Andersson, I., Hirsemann, H., Potdevin, G., Graafsma, H., Nilsson, B. & Spence, J. C. H. (2011). *Nature*, **470**, 73–77.

[bb14] Cheng, Q., Xu, C., Chai, J., Zhang, R., Wai Chi Chan, E. & Chen, S. (2020). *ACS Infect. Dis.* **6**, 577–587. 10.1021/acsinfecdis.9b00345 31709791

[bb15] Coquelle, N., Sliwa, M., Woodhouse, J., Schirò, G., Adam, V., Aquila, A., Barends, T. R. M., Boutet, S., Byrdin, M., Carbajo, S., De la Mora, E., Doak, R. B., Feliks, M., Fieschi, F., Foucar, L., Guillon, V., Hilpert, M., Hunter, M. S., Jakobs, S., Koglin, J. E., Kovacsova, G., Lane, T. J., Lévy, B., Liang, M. N., Nass, K., Ridard, J., Robinson, J. S., Roome, C. M., Ruckebusch, C., Seaberg, M., Thepaut, M., Cammarata, M., Demachy, I., Field, M., Shoeman, R. L., Bourgeois, D., Colletier, J.-P., Schlichting, I. & Weik, M. (2018). *Nat. Chem.* **10**, 31–37. 10.1038/nchem.2853 29256511

[bb16] Dashti, A., Mashayekhi, G., Shekhar, M., Ben Hail, D., Salah, S., Schwander, P., des Georges, A., Singharoy, A., Frank, J. & Ourmazd, A. (2020). *Nat. Commun.* **11**, 4734. 10.1038/s41467-020-18403-x PMC7501871 32948759

[bb17] Decking, W., Abeghyan, S., Abramian, P., Abramsky, A., Aguirre, A., Albrecht, C., Alou, P., Altarelli, M., Altmann, P., Amyan, K., Anashin, V., Apostolov, E., Appel, K., Auguste, D., Ayvazyan, V., Baark, S., Babies, F., Baboi, N., Bak, P., Balandin, V., Baldinger, R., Baranasic, B., Barbanotti, S., Belikov, O., Belokurov, V., Belova, L., Belyakov, V., Berry, S., Bertucci, M., Beutner, B., Block, A., Blocher, M., Bockmann, T., Bohm, C., Bohnert, M., Bondar, V., Bondarchuk, E., Bonezzi, M., Borowiec, P., Bosch, C., Bosenberg, U., Bosotti, A., Bospflug, R., Bousonville, M., Boyd, E., Bozhko, Y., Brand, A., Branlard, J., Briechle, S., Brinker, F., Brinker, S., Brinkmann, R., Brockhauser, S., Brovko, O., Bruck, H., Brudgam, A., Butkowski, L., Buttner, T., Calero, J., Castro-Carballo, E., Cattalanotto, G., Charrier, J., Chen, J., Cherepenko, A., Cheskidov, V., Chiodini, M., Chong, A., Choroba, S., Chorowski, M., Churanov, D., Cichalewski, W., Clausen, M., Clement, W., Cloue, C., Cobos, J. A., Coppola, N., Cunis, S., Czuba, K., Czwalinna, M., D’Almagne, B., Dammann, J., Danared, H., Wagner, A. D., Delfs, A., Delfs, T., Dietrich, F., Dietrich, T., Dohlus, M., Dommach, M., Donat, A., Dong, X., Doynikov, N., Dressel, M., Duda, M., Duda, P., Eckoldt, H., Ehsan, W., Eidam, J., Eints, F., Engling, C., Englisch, U., Ermakov, A., Escherich, K., Eschke, J., Saldin, E., Faesing, M., Fallou, A., Felber, M., Fenner, M., Fernandes, B., Fernandez, J. M., Feuker, S., Filippakopoulos, K., Floettmann, K., Fogel, V., Fontaine, M., Frances, A., Martin, I. F., Freund, W., Freyermuth, T., Friedland, M., Frohlich, L., Fusetti, M., Fydrych, J., Gallas, A., Garcia, O., Garcia-Tabares, L., Geloni, G., Gerasimova, N., Gerth, C., Gessler, P., Gharibyan, V., Gloor, M., Glowinkowski, J., Goessel, A., Golebiewski, Z., Golubeva, N., Grabowski, W., Graeff, W., Grebentsov, A., Grecki, M., Grevsmuehl, T., Gross, M., Grosse-Wortmann, U., Grunert, J., Grunewald, S., Grzegory, P., Feng, G., Guler, H., Gusev, G., Gutierrez, J. L., Hagge, L., Hamberg, M., Hanneken, R., Harms, E., Hartl, I., Hauberg, A., Hauf, S., Hauschildt, J., Hauser, J., Havlicek, J., Hedqvist, A., Heidbrook, N., Hellberg, F., Henning, D., Hensler, O., Hermann, T., Hidvegi, A., Hierholzer, M., Hintz, H., Hoffmann, F., Hoffmann, M., Hoffmann, M., Holler, Y., Huning, M., Ignatenko, A., Ilchen, M., Iluk, A., Iversen, J., Iversen, J., Izquierdo, M., Jachmann, L., Jardon, N., Jastrow, U., Jensch, K., Jensen, J., Dotabek, M. J. O., Jidda, M., Jin, H., Johansson, N., Jonas, R., Kaabi, W., Kaefer, D., Kammering, R., Kapitza, H., Karabekyan, S., Karstensen, S., Kasprzak, K., Katalev, V., Keese, D., Keil, B., Kholopov, M., Killenberger, M., Kitaev, B., Klimchenko, Y., Klos, R., Knebel, L., Koch, A., Koepke, M., Kohler, S., Kohler, W., Kohlstrunk, N., Konopkova, Z., Konstantinov, A., Kook, W., Koprek, W., Korfer, M., Korth, O., Kosarev, A., Kosinski, K., Kostin, D., Kot, Y., Kotarba, A., Kozak, T., Kozak, V., Kramert, R., Krasilnikov, M., Krasnov, A., Krause, B., Kravchuk, L., Krebs, O., Kretschmer, R., Kreutzkamp, J., Kroplin, O., Krzysik, K., Kube, G., Kuehn, H., Kujala, N., Kulikov, V., Kuzminych, V., La Civita, D., Lacroix, M., Lamb, T., Lancetov, A., Larsson, M., Le Pinvidic, D., Lederer, S., Lensch, T., Lenz, D., Leuschner, A., Levenhagen, F., Li, Y., Liebing, J., Lilje, L., Limberg, T., Lipka, D., List, B., Liu, J., Liu, S., Lorbeer, B., Lorkiewicz, J., Lu, H. H., Ludwig, F., Machau, K., Maciocha, W., Madec, C., Magueur, C., Maiano, C., Maksimova, I., Malcher, K., Maltezopoulos, T., Mamoshkina, E., Manschwetus, B., Marcellini, F., Marinkovic, G., Martinez, T., Martirosyan, H., Maschmann, W., Maslov, M., Matheisen, A., Mavric, U., Meissner, J., Meissner, K., Messer­schmidt, M., Meyners, N., Michalski, G., Michelato, P., Mildner, N., Moe, M., Moglia, F., Mohr, C., Mohr, S., Moller, W., Mommerz, M., Monaco, L., Montiel, C., Moretti, M., Morozov, I., Morozov, P., Mross, D., Mueller, J., Muller, C., Muller, J., Muller, K., Munilla, J., Munnich, A., Muratov, V., Napoly, O., Naser, B., Nefedov, N., Neumann, R., Neumann, R., Ngada, N., Noelle, D., Obier, F., Okunev, I., Oliver, J. A., Omet, M., Oppelt, A., Ottmar, A., Oublaid, M., Pagani, C., Paparella, R., Paramonov, V., Peitzmann, C., Penning, J., Perus, A., Peters, F., Petersen, B., Petrov, A., Petrov, I., Pfeiffer, S., Pfluger, J., Philipp, S., Pienaud, Y., Pierini, P., Pivovarov, S., Planas, M., Plawski, E., Pohl, M., Polinski, J., Popov, V., Prat, S., Prenting, J., Priebe, G., Pryschelski, H., Przygoda, K., Pyata, E., Racky, B., Rathjen, A., Ratuschni, W., Regnaud-Campderros, S., Rehlich, K., Reschke, D., Robson, C., Roever, J., Roggli, M., Rothenburg, J., Rusinski, E., Rybaniec, R., Sahling, H., Salmani, M., Samoylova, L., Sanzone, D., Saretzki, F., Sawlanski, O., Schaffran, J., Schlarb, H., Schlosser, M., Schlott, V., Schmidt, C., Schmidt-Foehre, F., Schmitz, M., Schmokel, M., Schnautz, T., Schneidmiller, E., Scholz, M., Schoneburg, B., Schultze, J., Schulz, C., Schwarz, A., Sekutowicz, J., Sellmann, D., Semenov, E., Serkez, S., Sertore, D., Shehzad, N., Shemarykin, P., Shi, L., Sienkiewicz, M., Sikora, D., Sikorski, M., Silenzi, A., Simon, C., Singer, W., Singer, X., Sinn, H., Sinram, K., Skvorodnev, N., Smirnow, P., Sommer, T., Sorokin, A., Stadler, M., Steckel, M., Steffen, B., Steinhau-Kuhl, N., Stephan, F., Stodulski, M., Stolper, M., Sulimov, A., Susen, R., Swierblewski, J., Sydlo, C., Syresin, E., Sytchev, V., Szuba, J., Tesch, N., Thie, J., Thiebault, A., Tiedtke, K., Tischhauser, D., Tolkiehn, J., Tomin, S., Tonisch, F., Toral, F., Torbin, I., Trapp, A., Treyer, D., Trowitzsch, G., Trublet, T., Tschentscher, T., Ullrich, F., Vannoni, M., Varela, P., Varghese, G., Vashchenko, G., Vasic, M., Vazquez-Velez, C., Verguet, A., Vilcins-Czvitkovits, S., Villanueva, R., Visentin, B., Viti, M., Vogel, E., Volobuev, E., Wagner, R., Walker, N., Wamsat, T., Weddig, H., Weichert, G., Weise, H., Wenndorf, R., Werner, M., Wichmann, R., Wiebers, C., Wiencek, M., Wilksen, T., Will, I., Winkelmann, L., Winkowski, M., Wittenburg, K., Witzig, A., Wlk, P., Wohlenberg, T., Wojciechowski, M., Wolff-Fabris, F., Wrochna, G., Wrona, K., Yakopov, M., Yang, B., Yang, F., Yurkov, M., Zagorodnov, I., Zalden, P., Zavadtsev, A., Zavadtsev, D., Zhirnov, A., Zhukov, A., Ziemann, V., Zolotov, A., Zolotukhina, N., Zummack, F. & Zybin, D. (2020). *Nat. Photonics*, **14**, 391–397.

[bb18] Dods, R., Båth, P., Morozov, D., Gagnér, V. A., Arnlund, D., Luk, H. L., Kübel, J., Maj, M., Vallejos, A., Wickstrand, C., Bosman, R., Beyerlein, K. R., Nelson, G., Liang, M. N., Milathianaki, D., Robinson, J., Harimoorthy, R., Berntsen, P., Malmerberg, E., Johansson, L., Andersson, R., Carbajo, S., Claesson, E., Conrad, C. E., Dahl, P., Hammarin, G., Hunter, M. S., Li, C. F., Lisova, S., Royant, A., Safari, C., Sharma, A., Williams, G. J., Yefanov, O., Westenhoff, S., Davidsson, J., DePonte, D. P., Boutet, S., Barty, A., Katona, G., Groenhof, G., Brändén, G. & Neutze, R. (2021). *Nature*, **589**, 310–314.

[bb19] Eriksson, M. (2016). *AIP Conf. Proc.* **1741**, 020001.

[bb20] Fair, R. J. & Tor, Y. (2014). *Perspect. Medicin. Chem.* **6**, PMC.S14459. 10.4137/PMC.S14459 PMC4159373 25232278

[bb21] Geremia, S., Campagnolo, M., Demitri, N. & Johnson, L. N. (2006). *Structure*, **14**, 393–400. 10.1016/j.str.2005.12.007 16531224

[bb22] Gevorkov, Y., Yefanov, O., Barty, A., White, T. A., Mariani, V., Brehm, W., Tolstikova, A., Grigat, R.-R. & Chapman, H. N. (2019). *Acta Cryst.* A**75**, 694–704. 10.1107/S2053273319010593 PMC6718201 31475914

[bb23] Gourinchas, G., Etzl, S., Göbl, C., Vide, U., Madl, T. & Winkler, A. (2017). *Sci. Adv.* **3**, e1602498. 10.1126/sciadv.1602498 PMC5336353 28275738

[bb24] Grünbein, M. L., Gorel, A., Foucar, L., Carbajo, S., Colocho, W., Gilevich, S., Hartmann, E., Hilpert, M., Hunter, M., Kloos, M., Koglin, J. E., Lane, T. J., Lewandowski, J., Lutman, A., Nass, K., Nass Kovacs, G., Roome, C. M., Sheppard, J., Shoeman, R. L., Stricker, M., van Driel, T., Vetter, S., Doak, R. B., Boutet, S., Aquila, A., Decker, F. J., Barends, T. R. M., Stan, C. A. & Schlichting, I. (2021). *Nat. Commun.* **12**, 1672. 10.1038/s41467-021-21819-8 PMC7960726 33723266

[bb25] Han, H., Round, E., Schubert, R., Gül, Y., Makroczyová, J., Meza, D., Heuser, P., Aepfelbacher, M., Barák, I., Betzel, C., Fromme, P., Kursula, I., Nissen, P., Tereschenko, E., Schulz, J., Uetrecht, C., Ulicný, J., Wilmanns, M., Hajdu, J., Lamzin, V. S. & Lorenzen, K. (2021). *J. Appl. Cryst.* **54**, 7–21. 10.1107/S1600576720013989 PMC7941304 33833637

[bb26] Hartmann, N., Helml, W., Galler, A., Bionta, M. R., Grünert, J. L., Molodtsov, S., Ferguson, K. R., Schorb, S., Swiggers, M. L., Carron, S., Bostedt, C., Castagna, J., Bozek, J., Glownia, J. M., Kane, D. J., Fry, A. R., White, W. E., Hauri, C. P., Feurer, T. & Coffee, R. N. (2014). *Nat. Photonics*, **8**, 706–709.

[bb27] Holtorf, H., Reinbothe, S., Reinbothe, C., Bereza, B. & Apel, K. (1995). *Proc. Natl Acad. Sci. USA*, **92**, 3254–3258. 10.1073/pnas.92.8.3254 PMC42144 7724548

[bb28] Hugonnet, J. E. & Blanchard, J. S. (2007). *Biochemistry*, **46**, 11998–12004. 10.1021/bi701506h PMC2593862 17915954

[bb29] Imming, P., Sinning, C. & Meyer, A. (2006). *Nat. Rev. Drug Discov.* **5**, 821–834. 10.1038/nrd2132 17016423

[bb30] Ishigami, I., Lewis-Ballester, A., Echelmeier, A., Brehm, G., Zatsepin, N. A., Grant, T. D., Coe, J. D., Lisova, S., Nelson, G., Zhang, S., Dobson, Z. F., Boutet, S., Sierra, R. G., Batyuk, A., Fromme, P., Fromme, R., Spence, J. C. H., Ros, A., Yeh, S. R. & Rousseau, D. L. (2019). *Proc. Natl Acad. Sci. USA*, **116**, 3572–3577. 10.1073/pnas.1814526116 PMC6397517 30808749

[bb31] Jeffrey, G. A. (1997). *An Introduction to Hydrogen Bonding.* Oxford University Press.

[bb32] Kern, J., Chatterjee, R., Young, I. D., Fuller, F. D., Lassalle, L., Ibrahim, M., Gul, S., Fransson, T., Brewster, A. S., Alonso-Mori, R., Hussein, R., Zhang, M., Douthit, L., de Lichtenberg, C., Cheah, M. H., Shevela, D., Wersig, J., Seuffert, I., Sokaras, D., Pastor, E., Weninger, C., Kroll, T., Sierra, R. G., Aller, P., Butryn, A., Orville, A. M., Liang, M., Batyuk, A., Koglin, J. E., Carbajo, S., Boutet, S., Moriarty, N. W., Holton, J. M., Dobbek, H., Adams, P. D., Bergmann, U., Sauter, N. K., Zouni, A., Messinger, J., Yano, J. & Yachandra, V. K. (2018). *Nature*, **563**, 421–425.

[bb33] Kostov, K. S. & Moffat, K. (2011). *Biophys. J.* **100**, 440–449. 10.1016/j.bpj.2010.10.053 PMC3021666 21244840

[bb34] Kupitz, C., Basu, S., Grotjohann, I., Fromme, R., Zatsepin, N. A., Rendek, K. N., Hunter, M. S., Shoeman, R. L., White, T. A., Wang, D., James, D., Yang, J.-H., Cobb, D. E., Reeder, B., Sierra, R. G., Liu, H., Barty, A., Aquila, A. L., Deponte, D., Kirian, R. A., Bari, S., Bergkamp, J. J., Beyerlein, K. R., Bogan, M. J., Caleman, C., Chao, T.-C., Conrad, C. E., Davis, K. M., Fleckenstein, H., Galli, L., Hau-Riege, S. P., Kassemeyer, S., Laksmono, H., Liang, M., Lomb, L., Marchesini, S., Martin, A. V., Messerschmidt, M., Milathianaki, D., Nass, K., Ros, A., Roy-Chowdhury, S., Schmidt, K., Seibert, M., Steinbrener, J., Stellato, F., Yan, L., Yoon, C., Moore, T. A., Moore, A. L., Pushkar, Y., Williams, G. J., Boutet, S., Doak, R. B., Weierstall, U., Frank, M., Chapman, H. N., Spence, J. C. H. & Fromme, P. (2014). *Nature*, **513**, 261–265.

[bb35] Kupitz, C., Olmos, J. L. Jr, Holl, M., Tremblay, L., Pande, K., Pandey, S., Oberthür, D., Hunter, M., Liang, M., Aquila, A., Tenboer, J., Calvey, G., Katz, A., Chen, Y., Wiedorn, M. O., Knoska, J., Meents, A., Majriani, V., Norwood, T., Poudyal, I., Grant, T., Miller, M. D., Xu, W., Tolstikova, A., Morgan, A., Metz, M., Martin-Garcia, J. M., Zook, J. D., Roy-Chowdhury, S., Coe, J., Nagaratnam, N., Meza, D., Fromme, R., Basu, S., Frank, M., White, T., Barty, A., Bajt, S., Yefanov, O., Chapman, H. N., Zatsepin, N., Nelson, G., Weierstall, U., Spence, J., Schwander, P., Pollack, L., Fromme, P., Ourmazd, A., Phillips, G. N. Jr & Schmidt, M. (2017). *Struct. Dyn.* **4**, 044003. 10.1063/1.4972069 PMC5178802 28083542

[bb36] Li, J., Liu, Z., Tan, C., Guo, X., Wang, L., Sancar, A. & Zhong, D. (2010). *Nature*, **466**, 887–890. 10.1038/nature09192 PMC3018752 20657578

[bb1] Liebschner, D., Afonine, P. V., Baker, M. L., Bunkóczi, G., Chen, V. B., Croll, T. I., Hintze, B., Hung, L.-W., Jain, S., McCoy, A. J., Moriarty, N. W., Oeffner, R. D., Poon, B. K., Prisant, M. G., Read, R. J., Richardson, J. S., Richardson, D. C., Sammito, M. D., Sobolev, O. V., Stockwell, D. H., Terwilliger, T. C., Urzhumtsev, A. G., Videau, L. L., Williams, C. J. & Adams, P. D. (2019). *Acta Cryst.* D**75**, 861–877. 10.1107/S2059798319011471 PMC6778852 31588918

[bb37] Liebschner, D., Afonine, P. V., Moriarty, N. W., Poon, B. K., Sobolev, O. V., Terwilliger, T. C. & Adams, P. D. (2017). *Acta Cryst.* D**73**, 148–157. 10.1107/S2059798316018210 PMC5297918 28177311

[bb38] Lomb, L., Barends, T. R. M., Kassemeyer, S., Aquila, A., Epp, S. W., Erk, B., Foucar, L., Hartmann, R., Rudek, B., Rolles, D., Rudenko, A., Shoeman, R. L., Andreasson, J., Bajt, S., Barthelmess, M., Barty, A., Bogan, M. J., Bostedt, C., Bozek, J. D., Caleman, C., Coffee, R., Coppola, N., DePonte, D. P., Doak, R. B., Ekeberg, T., Fleckenstein, H., Fromme, P., Gebhardt, M., Graafsma, H., Gumprecht, L., Hampton, C. Y., Hartmann, A., Hauser, G., Hirsemann, H., Holl, P., Holton, J. M., Hunter, M. S., Kabsch, W., Kimmel, N., Kirian, R. A., Liang, M., Maia, F. R. N. C., Meinhart, A., Marchesini, S., Martin, A. V., Nass, K., Reich, C., Schulz, J., Seibert, M. M., Sierra, R., Soltau, H., Spence, J. C. H., Steinbrener, J., Stellato, F., Stern, S., Timneanu, N., Wang, X., Weidenspointner, G., Weierstall, U., White, T. A., Wunderer, C., Chapman, H. N., Ullrich, J., Strüder, L. & Schlichting, I. (2011). *Phys. Rev. B*, **84**, 214111.

[bb39] Mancuso, A. P., Aquila, A., Batchelor, L., Bean, R. J., Bielecki, J., Borchers, G., Doerner, K., Giewekemeyer, K., Graceffa, R., Kelsey, O. D., Kim, Y., Kirkwood, H. J., Legrand, A., Letrun, R., Manning, B., Lopez Morillo, L., Messerschmidt, M., Mills, G., Raabe, S., Reimers, N., Round, A., Sato, T., Schulz, J., Signe Takem, C., Sikorski, M., Stern, S., Thute, P., Vagovič, P., Weinhausen, B. & Tschentscher, T. (2019). *J Synchrotron Rad*, **26**, 660–676. 10.1107/S1600577519003308 PMC6510195 31074429

[bb40] Mariani, V., Morgan, A., Yoon, C. H., Lane, T. J., White, T. A., O’Grady, C., Kuhn, M., Aplin, S., Koglin, J., Barty, A. & Chapman, H. N. (2016). *J. Appl. Cryst.* **49**, 1073–1080. 10.1107/S1600576716007469 PMC4886993 27275150

[bb41] Mehrabi, P., Schulz, E. C., Agthe, M., Horrell, S., Bourenkov, G., von Stetten, D., Leimkohl, J. P., Schikora, H., Schneider, T. R., Pearson, A. R., Tellkamp, F. & Miller, R. J. D. (2019). *Nat. Methods*, **16**, 979–982. 10.1038/s41592-019-0553-1 31527838

[bb42] Mehrabi, P., Schulz, E. C., Dsouza, R., Müller-Werkmeister, H. M., Tellkamp, F., Miller, R. J. D. & Pai, E. F. (2019). *Science*, **365**, 1167–1170. 10.1126/science.aaw9904 31515393

[bb43] Minor, W., Cymborowski, M., Otwinowski, Z. & Chruszcz, M. (2006). *Acta Cryst.* D**62**, 859–866. 10.1107/S0907444906019949 16855301

[bb44] Moffat, K. (2001). *Chem. Rev.* **101**, 1569–1582. 10.1021/cr990039q 11709992

[bb45] Murshudov, G. N., Skubák, P., Lebedev, A. A., Pannu, N. S., Steiner, R. A., Nicholls, R. A., Winn, M. D., Long, F. & Vagin, A. A. (2011). *Acta Cryst.* D**67**, 355–367. 10.1107/S0907444911001314 PMC3069751 21460454

[bb46] Nass, K. (2019). *Acta Cryst.* D**75**, 211–218. 10.1107/S2059798319000317 PMC6400258 30821709

[bb47] Neutze, R., Wouts, R., van der Spoel, D., Weckert, E. & Hajdu, J. (2000). *Nature*, **406**, 752–757. 10.1038/35021099 10963603

[bb48] Nogly, P., Weinert, T., James, D., Carbajo, S., Ozerov, D., Furrer, A., Gashi, D., Borin, V., Skopintsev, P., Jaeger, K., Nass, K., Båth, P., Bosman, R., Koglin, J., Seaberg, M., Lane, T., Kekilli, D., Brünle, S., Tanaka, T., Wu, W., Milne, C., White, T., Barty, A., Weierstall, U., Panneels, V., Nango, E., Iwata, S., Hunter, M., Schapiro, I., Schertler, G., Neutze, R. & Standfuss, J. (2018). *Science*, **361**, eaat0094. 10.1126/science.aat0094 29903883

[bb49] Olmos, J. L. Jr, Pandey, S., Martin-Garcia, J. M., Calvey, G., Katz, A., Knoska, J., Kupitz, C., Hunter, M. S., Liang, M., Oberthuer, D., Yefanov, O., Wiedorn, M., Heyman, M., Holl, M., Pande, K., Barty, A., Miller, M. D., Stern, S., Roy-Chowdhury, S., Coe, J., Nagaratnam, N., Zook, J., Verburgt, J., Norwood, T., Poudyal, I., Xu, D., Koglin, J., Seaberg, M. H., Zhao, Y., Bajt, S., Grant, T., Mariani, V., Nelson, G., Subramanian, G., Bae, E., Fromme, R., Fung, R., Schwander, P., Frank, M., White, T. A., Weierstall, U., Zatsepin, N., Spence, J., Fromme, P., Chapman, H. N., Pollack, L., Tremblay, L., Ourmazd, A., Phillips, G. N. Jr & Schmidt, M. (2018). *BMC Biol.* **16**, 59.

[bb50] Pande, K., Hutchison, C. D. M., Groenhof, G., Aquila, A., Robinson, J. S., Tenboer, J., Basu, S., Boutet, S., DePonte, D., Liang, M., White, T., Zatsepin, N., Yefanov, O., Morozov, D., Oberthuer, D., Gati, C., Subramanian, G., James, D., Zhao, Y., Koralek, J., Brayshaw, J., Kupitz, C., Conrad, C., Roy-Chowdhury, S., Coe, J. D., Metz, M., Xavier, P. L., Grant, T. D., Koglin, J. E., Ketawala, G., Fromme, R., Šrajer, V., Henning, R., Spence, J. C. H., Ourmazd, A., Schwander, P., Weierstall, U., Frank, M., Fromme, P., Barty, A., Chapman, H. N., Moffat, K., van Thor, J. J. & Schmidt, M. (2016). *Science*, **352**, 725–729.

[bb51] Pandey, S., Bean, R., Sato, T., Poudyal, I., Bielecki, J., Cruz Villarreal, J., Yefanov, O., Mariani, V., White, T. A., Kupitz, C., Hunter, M., Abdellatif, M. H., Bajt, S., Bondar, V., Echelmeier, A., Doppler, D., Emons, M., Frank, M., Fromme, R., Gevorkov, Y., Giovanetti, G., Jiang, M., Kim, D., Kim, Y., Kirkwood, H., Klimovskaia, A., Knoska, J., Koua, F. H. M., Letrun, R., Lisova, S., Maia, L., Mazalova, V., Meza, D., Michelat, T., Ourmazd, A., Palmer, G., Ramilli, M., Schubert, R., Schwander, P., Silenzi, A., Sztuk-Dambietz, J., Tolstikova, A., Chapman, H. N., Ros, A., Barty, A., Fromme, P., Mancuso, A. P. & Schmidt, M. (2020). *Nat. Methods*, **17**, 73–78. 10.1038/s41592-019-0628-z PMC9113060 31740816

[bb52] Ramakrishnan, S., Stagno, J. R., Conrad, C. E., Ding, J., Yu, P., Bhandari, Y. R., Lee, Y. T., Pauly, G., Yefanov, O., Wiedorn, M. O., Knoska, J., Oberthür, D., White, T. A., Barty, A., Mariani, V., Li, C., Brehm, W., Heinz, W. F., Magidson, V., Lockett, S., Hunter, M. S., Boutet, S., Zatsepin, N. A., Zuo, X., Grant, T. D., Pandey, S., Schmidt, M., Spence, J. C. H., Chapman, H. N. & Wang, Y.-X. (2021). *Nat. Commun.* **12**, 1762. 10.1038/s41467-021-21838-5 PMC7979858 33741910

[bb53] Ren, Z., Perman, B., Šrajer, V., Teng, T.-Y., Pradervand, C., Bourgeois, D., Schotte, F., Ursby, T., Kort, R., Wulff, M. & Moffat, K. (2001). *Biochemistry*, **40**, 13788–13801. 10.1021/bi0107142 11705368

[bb54] Schmidt, M. (2008). *Ultrashort Laser Pulses in Medicine and Biology*, edited by M. Braun, P. Gilch & W. Zinth, pp. 201–241. Berlin, Heidelberg, New York: Springer

[bb55] Schmidt, M. (2013). *Adv. Condens. Matter Phys.* **2013**, 1–10.

[bb56] Schmidt, M. (2020). *Crystals*, **10**, 116.

[bb57] Schmidt, M., Rajagopal, S., Ren, Z. & Moffat, K. (2003). *Biophys. J.* **84**, 2112–2129. 10.1016/S0006-3495(03)75018-8 PMC1302779 12609912

[bb58] Schmidt, M., Srajer, V., Henning, R., Ihee, H., Purwar, N., Tenboer, J. & Tripathi, S. (2013). *Acta Cryst.* D**69**, 2534–2542. 10.1107/S0907444913025997 PMC3852658 24311594

[bb59] Skopintsev, P., Ehrenberg, D., Weinert, T., James, D., Kar, R. K., Johnson, P. J. M., Ozerov, D., Furrer, A., Martiel, I., Dworkowski, F., Nass, K., Knopp, G., Cirelli, C., Arrell, C., Gashi, D., Mous, S., Wranik, M., Gruhl, T., Kekilli, D., Brünle, S., Deupi, X., Schertler, G. F. X., Benoit, R. M., Panneels, V., Nogly, P., Schapiro, I., Milne, C., Heberle, J. & Standfuss, J. (2020). *Nature*, **583**, 314–318. 10.1038/s41586-020-2307-8 32499654

[bb60] Smith, T., Wolff, K. A. & Nguyen, L. (2013). *Curr. Top. Microbiol. Immunol.* **374**, 53–80. 10.1007/82_2012_279 PMC3982203 23179675

[bb61] Sorigué, D., Hadjidemetriou, K., Blangy, S., Gotthard, G., Bonvalet, A., Coquelle, N., Samire, P., Aleksandrov, A., Antonucci, L., Benachir, A., Boutet, S., Byrdin, M., Cammarata, M., Carbajo, S., Cuiné, S., Doak, R. B., Foucar, L., Gorel, A., Grünbein, M., Hartmann, E., Hienerwadel, R., Hilpert, M., Kloos, M., Lane, T. J., Légeret, B., Legrand, P., Li-Beisson, Y., Moulin, S. L. Y., Nurizzo, D., Peltier, G., Schirò, G., Shoeman, R. L., Sliwa, M., Solinas, X., Zhuang, B., Barends, T. R. M., Colletier, J., Joffre, M., Royant, A., Berthomieu, C., Weik, M., Domratcheva, T., Brettel, K., Vos, M. H., Schlichting, I., Arnoux, P., Müller, P. & Beisson, F. (2021). *Science*, **372**, eabd5687. 10.1126/science.abd5687 33833098

[bb62] Sorigué, D., Légeret, B., Cuiné, S., Blangy, S., Moulin, S., Billon, E., Richaud, P., Brugière, S., Couté, Y., Nurizzo, D., Müller, P., Brettel, K., Pignol, D., Arnoux, P., Li-Beisson, Y., Peltier, G. & Beisson, F. (2017). *Science*, **357**, 903–907. 10.1126/science.aan6349 28860382

[bb63] Šrajer, V. & Schmidt, V. (2017). *J. Phys. D Appl. Phys.* **50**, 373001. 10.1088/1361-6463/aa7d32 PMC5771432 29353938

[bb64] Stagno, J. R., Liu, Y., Bhandari, Y. R., Conrad, C. E., Panja, S., Swain, M., Fan, L., Nelson, G., Li, C., Wendel, D. R., White, T. A., Coe, J. D., Wiedorn, M. O., Knoska, J., Oberthuer, D., Tuckey, R. A., Yu, P., Dyba, M., Tarasov, S. G., Weierstall, U., Grant, T. D., Schwieters, C. D., Zhang, J., Ferré-D’Amaré, A. R., Fromme, P., Draper, D. E., Liang, M., Hunter, M. S., Boutet, S., Tan, K., Zuo, X., Ji, X., Barty, A., Zatsepin, N. A., Chapman, H. N., Spence, J. C. H., Woodson, S. A. & Wang, Y.-X. (2017). *Nature*, **541**, 242–246. 10.1038/nature20599 PMC5502819 27841871

[bb65] Takala, H., Björling, A., Berntsson, O., Lehtivuori, H., Niebling, S., Hoernke, M., Kosheleva, I., Henning, R., Menzel, A., Ihalainen, J. A. & Westenhoff, S. (2014). *Nature*, **509**, 245–248. 10.1038/nature13310 PMC4015848 24776794

[bb66] Tassoni, R., Blok, A., Pannu, N. S. & Ubbink, M. (2019). *Biochemistry*, **58**, 997–1009. 10.1021/acs.biochem.8b01085 PMC6383187 30632739

[bb67] Tenboer, J., Basu, S., Zatsepin, N., Pande, K., Milathianaki, D., Frank, M., Hunter, M., Boutet, S., Williams, G. J., Koglin, J. E., Oberthuer, D., Heymann, M., Kupitz, C., Conrad, C., Coe, J., Roy-Chowdhury, S., Weierstall, U., James, D., Wang, D., Grant, T., Barty, A., Yefanov, O., Scales, J., Gati, C., Seuring, C., Srajer, V., Henning, R., Schwander, P., Fromme, R., Ourmazd, A., Moffat, K., Van Thor, J. J., Spence, J. C. H., Fromme, P., Chapman, H. N. & Schmidt, M. (2014). *Science*, **346**, 1242–1246. 10.1126/science.1259357 PMC4361027 25477465

[bb68] Totir, M. A., Helfand, M. S., Carey, M. P., Sheri, A., Buynak, J. D., Bonomo, R. A. & Carey, P. R. (2007). *Biochemistry*, **46**, 8980–8987. 10.1021/bi7006146 PMC2596720 17630699

[bb69] Tremblay, L. W., Xu, H. & Blanchard, J. S. (2010). *Biochemistry*, **49**, 9685–9687. 10.1021/bi1015088 PMC2981129 20961112

[bb70] Tzeng, S. R. & Kalodimos, C. G. (2012). *Nature*, **488**, 236–240. 10.1038/nature11271 22801505

[bb71] Vogt, A. D. & Di Cera, E. (2012). *Biochemistry*, **51**, 5894–5902. 10.1021/bi3006913 PMC3550001 22775458

[bb72] Wanzenberg, R., Agapov, I., Brefeld, W., Brinkmann, R., Chae, Y., Chao, H., Keil, J., Gavaldà, X. N., Röhlsberger, R., Schroer, C. G., Tischer, M. & Weckert, E. (2019). *AIP Conf. Proc.* **2054**, 030002.

[bb73] White, T. A., Mariani, V., Brehm, W., Yefanov, O., Barty, A., Beyerlein, K. R., Chervinskii, F., Galli, L., Gati, C., Nakane, T., Tolstikova, A., Yamashita, K., Yoon, C. H., Diederichs, K. & Chapman, H. N. (2016). *J. Appl. Cryst.* **49**, 680–689. 10.1107/S1600576716004751 PMC4815879 27047311

[bb74] Wiedorn, M. O., Awel, S., Morgan, A. J., Ayyer, K., Gevorkov, Y., Fleckenstein, H., Roth, N., Adriano, L., Bean, R., Beyerlein, K. R., Chen, J., Coe, J., Cruz-Mazo, F., Ekeberg, T., Graceffa, R., Heymann, M., Horke, D. A., Knoška, J., Mariani, V., Nazari, R., Oberthür, D., Samanta, A. K., Sierra, R. G., Stan, C. A., Yefanov, O., Rompotis, D., Correa, J., Erk, B., Treusch, R., Schulz, J., Hogue, B. G., Gañán-Calvo, A. M., Fromme, P., Küpper, J., Rode, A. V., Bajt, S., Kirian, R. A. & Chapman, H. N. (2018). *IUCrJ*, **5**, 574–584. 10.1107/S2052252518008369 PMC6126653 30224961

[bb75] Wiedorn, M. O., Oberthür, D., Bean, R., Schubert, R., Werner, N., Abbey, B., Aepfelbacher, M., Adriano, L., Allahgholi, A., Al-Qudami, N., Andreasson, J., Aplin, S., Awel, S., Ayyer, K., Bajt, S., Barák, I., Bari, S., Bielecki, J., Botha, S., Boukhelef, D., Brehm, W., Brockhauser, S., Cheviakov, I., Coleman, M. A., Cruz-Mazo, F., Danilevski, C., Darmanin, C., Doak, R. B., Domaracky, M., Dörner, K., Du, Y., Fangohr, H., Fleckenstein, H., Frank, M., Fromme, P., Gañán-Calvo, A. M., Gevorkov, Y., Giewekemeyer, K., Ginn, H. M., Graafsma, H., Graceffa, R., Greiffenberg, D., Gumprecht, L., Göttlicher, P., Hajdu, J., Hauf, S., Heymann, M., Holmes, S., Horke, D. A., Hunter, M. S., Imlau, S., Kaukher, A., Kim, Y., Klyuev, A., Knoška, J., Kobe, B., Kuhn, M., Kupitz, C., Küpper, J., Lahey-Rudolph, J. M., Laurus, T., Le Cong, K., Letrun, R., Xavier, P. L., Maia, L., Maia, F., Mariani, V., Messerschmidt, M., Metz, M., Mezza, D., Michelat, T., Mills, G., Monteiro, D. C. F., Morgan, A., Mühlig, K., Munke, A., Münnich, A., Nette, J., Nugent, K. A., Nuguid, T., Orville, A. M., Pandey, S., Pena, G., Villanueva-Perez, P., Poehlsen, J., Previtali, G., Redecke, L., Riekehr, W. M., Rohde, H., Round, A., Safenreiter, T., Sarrou, I., Sato, T., Schmidt, M., Schmitt, B., Schönherr, R., Schulz, J., Sellberg, J. A., Seibert, M. M., Seuring, C., Shelby, M. L., Shoeman, R. L., Sikorski, M., Silenzi, A., Stan, C. A., Shi, X., Stern, S., Sztuk-Dambietz, J., Szuba, J., Tolstikova, A., Trebbin, M., Trunk, U., Vagovic, P., Ve, T., Weinhausen, B., White, T. A., Wrona, K., Xu, C., Yefanov, O., Zatsepin, N., Zhang, J., Perbandt, M., Mancuso, A. P., Betzel, C., Chapman, H. & Barty, A. (2018). *Nat. Commun.* **9**, 4025.

[bb76] Winn, M. D., Ballard, C. C., Cowtan, K. D., Dodson, E. J., Emsley, P., Evans, P. R., Keegan, R. M., Krissinel, E. B., Leslie, A. G. W., McCoy, A., McNicholas, S. J., Murshudov, G. N., Pannu, N. S., Potterton, E. A., Powell, H. R., Read, R. J., Vagin, A. & Wilson, K. S. (2011). *Acta Cryst.* D**67**, 235–242. 10.1107/S0907444910045749 PMC3069738 21460441

[bb77] Yefanov, O., Mariani, V., Gati, C., White, T. A., Chapman, H. N. & Barty, A. (2015). *Opt. Express*, **23**, 28459–28470. 10.1364/OE.23.028459 PMC4646514 26561117

[bb78] Yefanov, O., Oberthür, D., Bean, R., Wiedorn, M. O., Knoska, J., Pena, G., Awel, S., Gumprecht, L., Domaracky, M., Sarrou, I., Lourdu Xavier, P., Metz, M., Bajt, S., Mariani, V., Gevorkov, Y., White, T. A., Tolstikova, A., Villanueva-Perez, P., Seuring, C., Aplin, S., Estillore, A. D., Küpper, J., Klyuev, A., Kuhn, M., Laurus, T., Graafsma, H., Monteiro, D. C. F., Trebbin, M., Maia, F. R. N. C., Cruz-Mazo, F., Gañán-Calvo, A. M., Heymann, M., Darmanin, C., Abbey, B., Schmidt, M., Fromme, P., Giewekemeyer, K., Sikorski, M., Graceffa, R., Vagovic, P., Kluyver, T., Bergemann, M., Fangohr, H., Sztuk-Dambietz, J., Hauf, S., Raab, N., Bondar, V., Mancuso, A. P., Chapman, H. & Barty, A. (2019). *Struct. Dyn.* **6**, 064702. 10.1063/1.5124387 PMC6892710 31832488

[bb79] Yun, J.-H., Li, X., Yue, J., Park, J.-H., Jin, Z., Li, C., Hu, H., Shi, Y., Pandey, S., Carbajo, S., Boutet, S., Hunter, M. S., Liang, M., Sierra, R. G., Lane, T. J., Zhou, L., Weierstall, U., Zatsepin, N. A., Ohki, M., Tame, J. R. H., Park, S. Y., Spence, J. C. H., Zhang, W., Schmidt, M., Lee, W. & Liu, H. (2021). *Proc. Natl Acad. Sci. USA*, **118**, e2020486118.

[bb80] Zaitsev-Doyle, J. J., Puchert, A., Pfeifer, Y., Yan, H., Yorke, B. A., Müller-Werkmeister, H. M., Uetrecht, C., Rehbein, J., Huse, N., Pearson, A. R. & Sans, M. (2019). *RSC Adv.* **9**, 8695–8699. 10.1039/c9ra00968j PMC9061760 35518684

